# Identification of More Feasible MicroRNA–mRNA Interactions within Multiple Cancers Using Principal Component Analysis Based Unsupervised Feature Extraction

**DOI:** 10.3390/ijms17050696

**Published:** 2016-05-10

**Authors:** Y-h. Taguchi

**Affiliations:** Department of Physics, Chuo University, 1-13-27 Kasuga, Bunkyo-ku, Tokyo 112-8551, Japan; tag@granular.com; Tel.: +81-3-3817-1791; Fax: +81-3-3817-1792

**Keywords:** principal component analysis, feature extraction, miRNA–mRNA interaction, hepatocellular carcinoma, non-small cell lung cancer, esophageal squamous cell carcinoma, prostate cancer, colorectal/colon cancer, breast cancer

## Abstract

MicroRNA(miRNA)–mRNA interactions are important for understanding many biological processes, including development, differentiation and disease progression, but their identification is highly context-dependent. When computationally derived from sequence information alone, the identification should be verified by integrated analyses of mRNA and miRNA expression. The drawback of this strategy is the vast number of identified interactions, which prevents an experimental or detailed investigation of each pair. In this paper, we overcome this difficulty by the recently proposed principal component analysis (PCA)-based unsupervised feature extraction (FE), which reduces the number of identified miRNA–mRNA interactions that properly discriminate between patients and healthy controls without losing biological feasibility. The approach is applied to six cancers: hepatocellular carcinoma, non-small cell lung cancer, esophageal squamous cell carcinoma, prostate cancer, colorectal/colon cancer and breast cancer. In PCA-based unsupervised FE, the significance does not depend on the number of samples (as in the standard case) but on the number of features, which approximates the number of miRNAs/mRNAs. To our knowledge, we have newly identified miRNA–mRNA interactions in multiple cancers based on a single common (universal) criterion. Moreover, the number of identified interactions was sufficiently small to be sequentially curated by literature searches.

## 1. Introduction

MicroRNA(miRNA) is short non-coding RNA with an approximate length of 22 nt. Its canonical function is to target specific messenger RNAs (mRNAs) and post-transcriptionally suppress their expression. miRNAs bind to the three prime untranslated region of target mRNAs and promote their degradation or interrupt their translation [1]. Although an individual miRNA typically targets multiple (often more than 100) mRNAs, it also specifically contributes to various biological processes, such as development and disease progression. Thus, the detection of miRNA–mRNA interactions is very important [2]. Unfortunately, effective methods for identifying such interactions are very limited. To detect mRNA–miRNA bindings experimentally, we must target the bindings with antibodies, remove the antibodies, then extract and sequence the mRNA/miRNA segments. Identifying every miRNA–mRNA interaction by this complicated and expensive process is unrealistic; moreover, many of the interactions are highly context-specific. Although miRNA–mRNA interactions can also be identified by computational methods, these are generally sequence-based and cannot accommodate the context-dependency of miRNA–mRNA bindings. Therefore, computational identifications inevitably include numerous false positives (FP). Given the context dependent nature of miRNA–mRNA binding, the computational identification ability of miRNA–mRNA interactions can be greatly improved by accounting for the gene expression/miRNA expression. A potential drawback of this strategy is the vast number of possible mRNA–miRNA pairs. If (as is generally thought) each miRNA targets up to 30% of the mRNAs, *N* mRNAs in the presence of *M* miRNAs can form up to NM pairs. Given that typical values of *N* and *M* comprise a few tens of thousands and a few thousands, respectively, the number of possible pairs reaches several million. This suggests that miRNA and mRNA expressions are extremely well correlated, with *p*-values as small as $10^{-9}$, and are undetectable in noisy biological datasets. However, these difficulties could be reduced by reducing the number of pairs in the investigation.

Following this strategy, the present state-of-the-art analysis identifies differently expressed miRNAs and mRNAs whose interaction significance is supposed to be tested. Despite the relative success of this methodology, the number of candidate interacting mRNAs and miRNAs remains prohibitively high (see following discussion), preventing us from investigating individual mRNA–miRNA pairs and validating them experimentally.

The recently proposed principal component analysis (PCA)-based feature extraction (FE) can identify a small number of mRNAs/miRNAs associated with the differential reciprocal expression between distinct treatments or conditions. For example, Taguchi *et al.* [3] recently applied PCA-based unsupervised FE to heart diseases mediated by post-traumatic stress disorder, and identified distinct differential mRNA and miRNA expression among different treatments. We also reported targeting of the mRNAs by miRNAs, and a reciprocal correlation between the miRNAs and mRNAs. In the present study, I apply the PCA-based FE methodology to identify interacting mRNAs and miRNAs. This strategy successfully identified a limited number of miRNA–mRNA pairs whose interactions have been experimentally confirmed in previous studies, allowing a comprehensive literature search of each pair.

## 2. Results and Discussion

### 2.1. Hepatocellular Carcinoma (HCC)

Within the HCC dataset, between 269 mRNA probes and 58 miRNA probes identified as outliers (see Table 1), we have successfully reduced the number of identified miRNA–mRNA pairs (21 pairs, see Tables S1 and S2). Previous reports confirmed that almost all of these pairs are related to HCC. Many of these pairs are also listed in starbase [4] (see Materials and Methods and supplementary Tables). The number of miRNAs associated with significant reciprocal correlations in statbase is typically half the total number of candidate miRNAs (100 *versus* several hundred), supporting the suitability of our strategy. Especially, the small number of FPs demonstrates the core advantage of our strategies.

### 2.2. Non-Small Cell Lung Cancer (NSCLC)

In the NSCLC dataset, between 1098 mRNA probes and 268 miRNA probes identified as outliers (see Table 1), we identified a limited number of miRNA–mRNA pairs (311 pairs, see Tables S3–S12). These numbers are relatively large, because multiple probes are attributed to each mRNA and miRNA; that is, the total number of probes exceeds the total number of mRNAs and miRNAs, thereby reducing the numbers of outlier mRNAs and miRNAs might not be easy. Almost all of the identified miRNA–mRNA pairs have been documented in previous reports of NSCLC, and some were experimentally validated. BCL11A is reportedly regulated by miR-30a [5]. Moreover, a significant number of the miRNA–mRNA pairs are included in starbase (see supplementary Tables listed in the above). Again, the small number of FPs demonstrates the feasibility of our strategies.

### 2.3. Esophageal Squamous Cell Cancer (ESCC)

In the ESCC dataset, between 189 mRNA probes and 37 miRNA probes identified as outliers (see Table 1), we again successfully identified a limited number of miRNA–mRNA pairs (4 pairs, see Table S13). Although the number of identified pairs was very small, all of the candidate pairs had been previously reported to be associated with ESCC. Moreover, a significantly large number of miRNA–mRNA pairs were also included in starbase (see supplementary Tables listed in the above).

### 2.4. Prostate Cancer

Between 399 mRNA probes and 23 miRNA probes identified as outliers (see Table 1) within Prostate cancer data set, we again identified a limited number of miRNA–mRNA pairs (32 pairs, see Tables S14 and S15). However, the proportion of candidate interactions conclusively related to prostate cancer was smaller than in the other cancer datasets. One possible reason is the relative lack of interest in prostate cancer, which is less lethal than the other five cancers. This suggests that the identified interactions could be validated in future. Again, a significantly large number of the candidate miRNA–mRNA pairs were included in starbase (see supplementary Tables listed in the above), supporting that the small number of identified miRNA–mRNA pairs reported in the literature is due to the small number of studies.

### 2.5. Colorectal/Colon Cancer

In the colorectal/colon cancer data set, we identified 309 mRNA probes and 12 miRNA probes as outliers (see Table 1). The number of miRNA–mRNA pairs was successfully limited (8 pairs, see Table S16). Almost all of the candidate miRNA–mRNA pairs are mentioned in previous reports of colorectal/colon cancer, and a significantly large number of them are included in starbase. Again, these results support the feasibility of our strategy (see supplementary Tables listed in the above).

### 2.6. Breast Cancer

Finally, in the breast cancer dataset, we identified 980 mRNA probes and 18 miRNA probes as outliers (see Table 1). A limited number of miRNA–mRNA pairs were also identified (37 pairs, see Tables S17 and S18). Moreover, almost all of the identified pairs have been previously reported in the breast cancer literature, and some have been experimentally validated. In particular, miR-143 and 145 are known to synergistically regulate cell proliferation and invasion in breast cancer [6]. Again, a significantly large number of the identified miRNA–mRNA pairs are included in starbase (see supplementary Tables listed in the above), suggesting that our strategy is feasible for this database also.

### 2.7. Confirmation of Significance of the FDR Criterion

To clarify the feasibility of our mRNA–miRNA identification criterion, we compared the significance of our criterion with that of another well-known criterion, the false discovery rate (FDR; see methods). Table 2 compares the number of significant mRNAs/miRNAs in the FDR and Benjamini-Hochberg [7] (BH) criteria (histograms of the *p*-values and some additional displays provided by fdrtool are available as supplementary Figures). For all six cancers, the numbers of significant mRNAs were consistent between the two criteria (and were identical for prostate cancer and ESCC). This confirms that PCA-based unsupervised FE can identify the mRNAs involved in various cancers.

However, the significant miRNA identification largely differed between the BH and FDR criteria. The numbers of significant miRNAs were consistent only in colon/colorectal cancer. In three cancers (NSCLC, ESCC and breast cancer), FDR failed to identify any significant miRNAs. However, in these cancers, fewer than 10% of all miRNAs in each microarray were identified as significant by PCA-based unsupervised FE. Thus, such small numbers of miRNAs are difficult to identify correctly. Nevertheless, miRNAs/mRNAs identification by PCA-based unsupervised FE appears to be feasible in practice.

### 2.8. Discrimination Performance between Patients and Healthy Controls

Before discussing the identified miRNA–mRNA pairs, we demonstrate the feasibility of miRNA/mRNA identification by PCA-based unsupervised FE (see methods). To this end, we performed a discrimination analysis between patients and healthy controls (Table 3), using only the miRNAs/mRNAs identified by PCA-based unsupervised FE (see Table 1). The discrimination was obviously successful; the *p*-values were very small while the odds ratios were very large (exceeding 10, and often exceeding 100) for a small number of (a few) PC loadings. Undoubtedly, PCA-based unsupervised FE effectively reduces the number of critical miRNAs/mRNAs without optimizing the criteria for specific cancers. The valuable miRNA–mRNA pairs among the identified miRNAs and mRNAs are listed in Table 1.

### 2.9. Confident Candidate Selection by PCA-Based Unsupervised FE

From a methodological viewpoint, the identification of miRNA–mRNA interactions is limited by the large number of candidate pairs. Each miRNA targets approximately 30% of the mRNAs. Therefore, the number of candidate pairs is proportional to the product of the number of mRNAs and the number of miRNAs associated with significant differential expression between the controls and treated samples.

For example, in their study of miRNA–mRNA pairs in HCC, Ding *et al.* [8] identified several hundred miRNAs and a few thousand mRNAs that are differently expressed between normal tissues and tumors (FDR ≤0.01; $\log_{2}$ fold change ≥1).

Ma *et al.* [9], Zhang *et al.* [10] and Ma *et al.* [11] reported miRNA–mRNA interactions in NSCLC. In the first and second of these studies, the number of mRNAs (miRNAs) with different expression levels in normal tissues and tumors was 249 (90) and a few thousand (a few hundred), respectively. Ma *et al.* [11] identified 581 up-regulated and 1297 down-regulated mRNAs, as well as 25 up-regulated and 24 down-regulated miRNAs, that are differently expressed between normal tissues and tumors (FDR <0.1 by SAM, version 3.11; Stanford University, Stanford, CA, USA).

Wu *et al.* [12] analyzed the miRNA–mRNA interaction network in ESCC. They identified 56 miRNAs that are differently expressed in tumors and normal tissues. They also identified 35,942 significant (1.5-fold mRNA expression difference) miRNA–mRNA pairs in a combined expression analysis and *in silico* mRNA target inference. Yang *et al.* [13] identified 17 miRNAs that were differently expressed between tumor and normal tissues (FDR < 0.05). They also identified 576 upregulated probes and 1094 downregulated probes in ESCC samples (Fold change > 3; FDR < 0.001). Meng *et al.* [14] reported four differently expressed miRNAs in ESCC tumor samples and normal tissues (FDR < 0.05), and 1110 differentially expressed genes (516 and 594 with decreased and increased expression, respectively, relative to their normal counterparts; FDR < 0.05).

Zhang *et al.* [15] investigated miRNA–mRNA interactions in prostate cancer, and found correlations between the miRNAs and mRNAs (BH criterion adjusted p<0.008 or <0.065). However, the vast number of possible interactions prevented a direct analysis.

Fu *et al.* [16] investigated miRNA–mRNA interactions in colorectal cancer, and reported 32 differentially expressed miRNAs and 2916 mRNAs in CRC samples and their corresponding normal epithelial tissues (FDR < 0.05). Regarding miRNA–mRNA interactions in colon cancer, Li *et al.* [17] identified 31 down-regulated and 2 up-regulated miRNAs, and 73 up-regulated and 63 down-regulated mRNAs (>1.2-fold change; FDR < 0.1).

Bleckmann *et al.* [18] reported miRNA–mRNA interactions in breast cancer. Ninety-six of their identified miRNAs were not only differentially expressed in normal and cancer tissues, but also consistently regulated the target mRNA sets. However, the number of differentially expressed mRNAs was not mentioned.

Besides the above mentioned cancers, miRNA–mRNA interactions have been reported in various other cases. For example, Liu *et al*. [19] searched for miRNA–mRNA interactions by a state-of-the-art bioinformatics strategy. They listed as many as 620 mRNAs and 48 miRNAs that were significantly associated with differential expression between pancreatic ductal adenocarcinoma and normal tissues (*t* test and the Bonferroni’s correction- adjusted *p* value <0.05; ∣logFC∣ value >1). From these results, they identified 224 miRNA–mRNA interactions, and successfully integrated them into a network representation. However, their analysis could not reveal the progression of pancreatic ductal adenocarcinoma. This limitation is by no means rare. As another example, Zhuang *et al*. [20] identified 217 miRNAs and 791 miRNAs that were significantly enriched in downregulated and upregulated genes in non-obstructive azoospermia. They also found 2461 mRNA targets of 184 miRNAs (BH criterion [7], adjusted *p*-values <0.05 for fold change >2 or < 1/2). However, we could not compare their results with those of other studies, because the identified miRNA–mRNA interactions were too numerous.

In contrast to these state-of-the-art methodologies, our methodology identified a manageable number of miRNA/mRNA pairs, many of which had been experimentally validated in previous studies. Existing state-of-the-art methodologies inherently identify numerous mRNA/miRNA probes, because their *p*-values rely on the sample size. As the sample number grows, the *p*-values generally decrease but the number of significant up/downregulated mRNA/miRNA probes becomes unmanageable. The number of probes can be reduced to a treatable level by tuning the *p*-values (or fold changes). Obviously, the *p*-values and fold changes vary among studies, and even within the same study, which biases the analysis. In contrast, the number of probes in our outlier identification is independent of the number of samples. Thus, the PCA-based unsupervised FE is less sensitive to sample number than the existing methods. This might explain the successful identification of reasonable (treatable) numbers of genes, whose integrities were validated in an extensive literature search. To further validate the feasibility of the selected miRNAs/mRNAs, we employed them as biomarkers that can well discriminate between patients and healthy controls. To our knowledge, no previous miRNA–mRNA identifications have undertaken this kind of independent validation, because the biomarker identification problem is itself a difficult task, and independent of identifying miRNA–mRNA interactions.

More remarkably, our miRNA–mRNA identification was based on conserved sets provided by TargetScan, which contains mostly feasible pairs but also large numbers of false negatives (FN). Other studies have avoided the TargetScan algorithm because it yields insufficient numbers of miRNA–mRNA pairs. However, our methodology identifies feasible pairs in TargetScan data alone, suggesting its superior effectiveness to existing state-of-the-art methodologies.

The high ratio of confidently identified miRNA–mRNA pairs is a distinct advantage of our methodology. In existing approaches, the identified miRNA–mRNA pairs must be validated in further (often experimental) study. Thus, our methodology is very promising for identifying miRNA–miRNA interactions in gene expression datasets.

In all six cancers, significantly large numbers of the identified miRNA–mRNA pairs were also included in starbase. This further strengthens our methodology against existing approaches, which cannot be compared with existing databases because of the prohibitively many miRNA–mRNA pairs, precluding a manual evaluation.

### 2.10. Usefulness of Unmatched Data and Number of False Negatives

Before closing this section, I would like to discuss two topics that may be concerned. The first topic is the usage of unmatched data; all of analyses performed in this study employed unmatched data set between mRNAs and miRNAs. It is true that employing unmatched data can decrease the feasibility of the results than employing matched data. However, employing unmatched data has the great advantages that matched data can rarely fulfill; employing unmatched data allows us to consider more samples. mRNA/miRNA profiles in Table 1 were selected so as to have as many as samples. If it was restricted to matched data set, we could not consider as many as samples in Table 1. In addition to this, there were multiple published studies of miRNA–mRNA interaction employing unmatched data set [10,12,13,14,21]. Thus, using unmatched data solely cannot be the reason why the study should not be performed. The second topic is the number of FNs, *i.e.*, that of overlooked pairs. As I have emphasized in the above, the main purpose of this study is to identify more trustable pairs. This strategy inevitably results in the numerous FNs, since there are trade-off between number of FP and that of FNs. Trying to have less FP often results in more FNs. However, in this particular study FP is severer than FN. Since the number of gene expression profiles available in the public repositories will continuously increase, more studies can be performed, which will allow us to identify overlooked FNs. However, it is not easy to identify FPs by additional study, since it is impossible for us to distinguish the two situations; lacks in additional study is because of FP in the first study, or it is simply because of fluctuation. In this regard, I believe that minimization of FPs is more important than that of FNs, which motivated me to start this study. Thus, numerous possible FNs should not be the reason why this kind of studies should not be performed.

## 3. Materials and Methods

### 3.1. Gene Expression Profiles

We downloaded multiple mRNA/miRNA expression profiles of various cancer diseases from the gene expression omnibus (GEO) as follows. The expression profiles are summarized in Table 1, and detailed in the following subsections. As the miRNA expressions of colon cancer and breast cancer were $\log_{2}$ transformed, they were reconverted to their raw values before further analyses.

#### 3.1.1. HCC

The mRNA [22] and miRNA [23] expression profiles of HCC, also known as liver cancer, were downloaded from GEO using GEO ID GSE45114 (CapitalBio Human 22k oligonucleotide microarray) and GSE36915 (Illumina Human v2 MicroRNA expression beadchip), respectively. GSE45114_series_matrix.txt.gz and GSE36915_series_matrix.txt.gz were used and normalized to yield sample profiles with zero means and unit variances. No further normalizations were applied, as the profiles had already been normalized by the original researchers.

#### 3.1.2. NSCLC

The mRNA [24] and miRNA [25] expression profiles of NSCLC were downloaded from GEO using GEO ID GSE18842 (Affymetrix Human Genome U133 Plus 2.0 Array) and GSE15008 (National Engineering Research Center mammalian microRNA microarray), respectively. GSE15008_series_matrix.txt.gz and GSE18842_series_matrix.txt.gz were used and normalized to yield sample profiles with zero means and unit variances. No further normalizations were applied, as the profiles had already been normalized by the original researchers.

#### 3.1.3. ESCC

The mRNA [26] and miRNA [27] expression profiles of ESCC were downloaded from GEO using GEO ID GSE38129 (Affymetrix Human Genome U133A 2.0 Array) and GSE13937 (OSU-CCC Human and Mouse MicroRNA Microarray Version 3.0), respectively. GSE38129_series_matrix.txt.gz and the gpr files in GSE13937_RAW were used for mRNA and miRNA expressions, respectively. Because no miRNAs were identified in the GSE13937_series_matrix.txt.gz provided by the authors, we extracted the F635 Mean signals from the individual gpr files. The mRNA/miRNA expression profiles were normalized to give sample profiles with zero means and unit variances. No further normalizations were applied, as the mRNA profiles had already been normalized by the original researchers, whereas the miRNAs could be successfully identified without further normalization.

#### 3.1.4. Prostate Cancer

The mRNA [28] and miRNA [29] profiles of prostate cancer were downloaded from GEO using GEO ID GSE21032 (Affymetrix Human Exon 1.0 ST Array [probe set (exon) version]) and GSE64318 (Agilent-019118 Human miRNA Microarray 2.0 G4470B), respectively. The GSE21032_series_matrix.txt.gz and GSE64318_series_matrix.txt.gz files were used and normalized to yield sample profiles with zero means and unit variances. No further normalizations were applied, as the profiles had already been normalized by the original researchers.

#### 3.1.5. Colorectal/Colon Cancer

The mRNA [30] profiles of colorectal cancer and the miRNA [31] profiles of colon cancer were downloaded from GEO using GEO ID GSE41258 (Affymetrix Human Genome U133A Array) and GSE48267 (Agilent-021827 Human miRNA Microarray (V3) [miRBase release 12.0 miRNA ID version]), respectively. GSE41258_series_matrix.txt.gz and GSE48267_series_matrix.txt.gz were used and normalized to yield sample profiles with zero means and unit variances. No further normalizations were applied, as the profiles had already been normalized by the original researchers In the miRNA expression profiles (GSE48267), only the parafilm samples were analyzed because the PCA-based unsupervised FE identified no significant miRNAs in the snap files.

#### 3.1.6. Breast Tumors

The mRNA and miRNA expression profiles [32] of breast tumors were downloaded from GEO using GEO ID GSE29174 (NKI-CMF Homo sapiens 35k oligo array) and GSE29173 (Illumina Genome Analyzer IIx [Homo sapiens]). GSE28884-GPL3676_series_matrix.txt.gz and the files whose names end by “geo.txt” in GSE29173_RAW were used for mRNA and miRNA expressions, respectively. Reads annotated as individual miRNAs were summed over all expression levels of each miRNA. The mRNA/miRNA expression profiles were normalized to yield sample profiles with zero means and unit variances. No further normalizations were applied, as the mRNA profiles had already been normalized by the original researchers, whereas the miRNA profiles could be successfully identified without further normalization.

### 3.2. PCA-Based Unsupervised FE

PCA-based unsupervised FE has been extensively applied to various biological problems [3,33,34,35,36,37,38,39,40,41,42,43,44,45], but warrants a brief summary here. Let $x_{ij}$ be the expression of the *i*th mRNA/miRNA of the *j*th sample, and suppose that $\frac{1}{N(M)}\sum_{i}x_{ij}=0$ and $\frac{1}{N(M)}\sum_{i}x_{ij}^{2}=1$ where N(M) is the total number of mRNAs(miRNAs). The elements $x_{ij}$ are contained in a matrix *X*. In contrast to standard PCA, which embeds the samples, PCA- based unsupervised FE embeds the genes (miRNAs or mRNAs). Then *k*th principal component (PC) score $u_{ki}$ attributed to the *i*th gene is computed as an element of the eigenvector $u_{k}$ of the gram matrix $G\equiv XX^{T}$,

$$XX^{T}u_{k}=\lambda_{k}u_{k},$$

where the eigenvalues $\lambda_{k}$ are ordered such that $\lambda_{k+1}<\lambda_{k}$. Because we have

$$X^{T}Xv_{k}=X^{T}XX^{T}u_{k}=X^{T}\lambda_{k}u_{k}=\lambda_{k}v_{k},$$

the *k*th PC loading $v_{kj}$ attributed to the *j*th sample is computed as an element of $v_{k}=X^{T}u_{k}$, which is an eigenvector of the matrix $X^{T}X$. After identifying a set $\Omega_{k}$ of PCs with distinctly different loadings between tumors and normal tissues (*t* test, p<0.05), the outlier genes are identified by a *χ* squared distribution, assuming a Gaussian distribution of the PC scores:

(1) $$P_{i}=P\left[\sum_{k\in \Omega_{k}}{\left(\frac{u_{ki}}{\sigma_{k}}\right)}^{2}>x\right]$$

where P[>x] is the cumulative probability of the *χ* squared distribution, where the argument exceeds *x* and $\sigma_{k}$ is the standard deviation of the *k*th PC scores. Then, if the BH criterion [7]-adjusted $P_{i}$ is below 0.01, gene *i* is identified as an outlier.

### 3.3. Identification of Significant miRNA–mRNA Pairs

Some of the mRNA/miRNAs selected as outliers by the PCA-based unsupervised FE showed significant up/downregulation between normal control tissues and tumors (BH criterion [7]-adjusted p<0.05, *t* test). The list of conserved target genes of each miRNA was then obtained from TargetScan [46], and the miRNA–mRNA pairs associated with reciprocal regulation and identified by TargetScan were selected.

### 3.4. Validation Using Starbase

To further confirm the feasibility of “outlier” miRNA–mRNA pairs, we checked whether those pairs are associated with significant reciprocal correlations of their expression profiles in starbase [4], which includes 14 cancer datasets collected from multiple data sources. The cancers associated with significant reciprocal correlations of the outlier miRNA–mRNA pairs were counted and listed in the last column of the table of identified miRNA–mRNA pairs (Tables S1–S18).

### 3.5. Discrimination between Patients and Healthy Controls

Discrimination was performed by linear discriminant analysis (LDA) using PCA [40,41,42]; The LDA was performed by the lda function in R [47]. In this analysis, the PC loadings were recomputed using only the mRNAs or miRNAs selected by the PCA-based unsupervised FE (Table 1). The recomputed loadings were then attributed to samples. The leave-one-out cross validation was employed since we set CV = T. We also weighted both classes equally by setting prior = rep(1/2,2). The first *L* PC loadings were used for discrimination, and the optimal *L* for each cancer was found by trial-and-error. Fisher test was performed using fihser.test function in R [47].

### 3.6. Validation Using FDR

FDR was computed by the fdrtool function in the fdrtool package [48] in R [47]. The *p*-values were computed assuming the *χ* squared distribution (see Equation (1)), then were imported to fdrtool function with the option of staistic=“pvalue”. If the *q*-value computed by fdrtool was less than 0.01, the mRNA/miRNA was regarded as significant.

## 4. Conclusions

This paper proposes the application of PCA-based unsupervised FE to the identification of feasible miRNA–mRNA interactions. Based on an integrated analysis of mRNA and miRNA expression, the technique successfully limited the number of feasible interactions under a single criterion that is independent of disease type, number of samples and microarrays used. The methodology presents as an efficient approach for identifying miRNA–mRNA interactions in mRNA/miRNA gene expression data.

## Figures and Tables

**Table 1:** Summary of the investigated mRNA/miRNA expressions. Probes identified and not identified by PCA-based unsupervised FE are denoted as selected and non-selected, respectively. For more details, see Materials and Methods.

		Number of Samples		Number of Probes	
Cancers	GEO ID	Tumors	Controls	Selected	Non-Selected
HCC					
mRNA	GSE45114	24	25	269	22,963
miRNA	GSE36915	68	21	58	1087
NSCLC					
mRNA	GSE18842	46	45	1098	53,504
miRNA	GSE15008	187	174	268	3428
ESCC					
mRNA	GSE38129	30	30	189	22,088
miRNA	GSE19337	76	76	37	1217
Prostate cancer					
mRNA	GSE21032	150	29	399	43,020
miRNA	GSE84318	27	27	23	700
Colon/colorectal cancer					
mRNA	GSE41258	186	54	309	21,974
miRNA	GSE48267	30	30	12	839
Breast cancer					
mRNA	GSE29174	110	11	980	33,600
miRNA	GSE28884	173	16	18	2258

**Table 2:** Comparison between BH-adjusted *p*-values and FDR *q*-values. Under both criteria, mRNA/miRNA identifications with *p*-values below 0.01 were regarded as significant.

	HCC		NSCLC		ESCC	
	mRNA	miRNA	mRNA	miRNA	mRNA	miRNA
FDR	262	38	978	0	189	0
BH	269	58	1091	268	189	37
	**Prostate Cancer**		**Colon/Colorectal Cancer**		**Breast Cancer**	
	**mRNA**	**miRNA**	**mRNA**	**miRNA**	**mRNA**	**miRNA**
FDR	399	7	305	12	861	0
BH	399	23	309	12	908	18

**Table 3:** Results (confusion matrix) of linear discriminant analysis between patients and healthy controls (hc). *L*: number of PC loadings used in the discrimination; the *p*-values and odds ratios were computed by Fisher’s exact test. *: $p<2.22\times 10^{-16}$. Columns: true classes, rows: predicted classes.

	mRNA		miRNA	
	HCC	hc	HCC	hc
HCC	20	0	64	0
hc	4	25	4	21
(*L*, *p*-value, odds ratio)	(4, $3.75\times 10^{-10}$, *∞*)		(10, *, *∞*)	
	NSCLC	hc	NSCLC	hc
NSCLC	46	0	171	12
hc	0	45	16	162
(*L*, *p*-value, odds ratio)	(2,*, *∞*)		(5, *, $1.39\times 10^{2}$)	
	ESCC	hc	ESCC	hc
ESCC	28	2	63	11
hc	2	28	13	65
(*L*, *p*-value, odds ratio)	(2, $3.22\times 10^{-12}$, $1.54\times 10^{2}$)		(6, *, 2.77×10)	
	Pancreatic cancer	hc	Pancreatic cancer	hc
Pancreatic cancer	139	4	22	3
hc	11	25	5	24
(*L*, *p*-value, odds ratio)	(8, *, 7.45×10)		(4, $2.88\times 10^{-7}$, 3.17×10)	
	Colorectal cancer	hc	Colon cancer	hc
Colon/Colorectal cancer	178	5	27	3
hc	8	49	3	27
(*L*, *p*-value, odds ratio)	(8, *, $2.02\times 10^{2}$)		(4, $2.82\times 10^{-10}$, 6.98×10)	
	Breast cancer	hc	Breast cancer	hc
Breast cancer	110	0	169	5
hc	0	11	4	11
(*L*, *p*-value, odds ratio)	(3, $7.83\times 10^{-16}$, *∞*)		(18, $2.62\times 10^{-11}$, 8.49×10)	

## Supplementary Materials

Supplementary materials can be found at http://www.mdpi.com/1422-0067/17/ 5/696/s1.
