# Supplementary material for: Identification of More Feasible MicroRNA–mRNA Interactions within Multiple Cancers Using Principal Component Analysis Based Unsupervised Feature Extraction
Source: Int J Mol Sci. 2016 May 10;17(5):696. doi: 10.3390/ijms17050696 (PMC4881522; doi:10.3390/ijms17050696)
Supplement: Supplementary file 1 [file ijms-17-00696-s001.pdf]

Supplementary Materials: Identification of More Feasible MicroRNA–mRNA Interactions within Multiple Cancers Using Principal Component Analysis Based Unsupervised Feature Extraction

Y-h. Taguchi

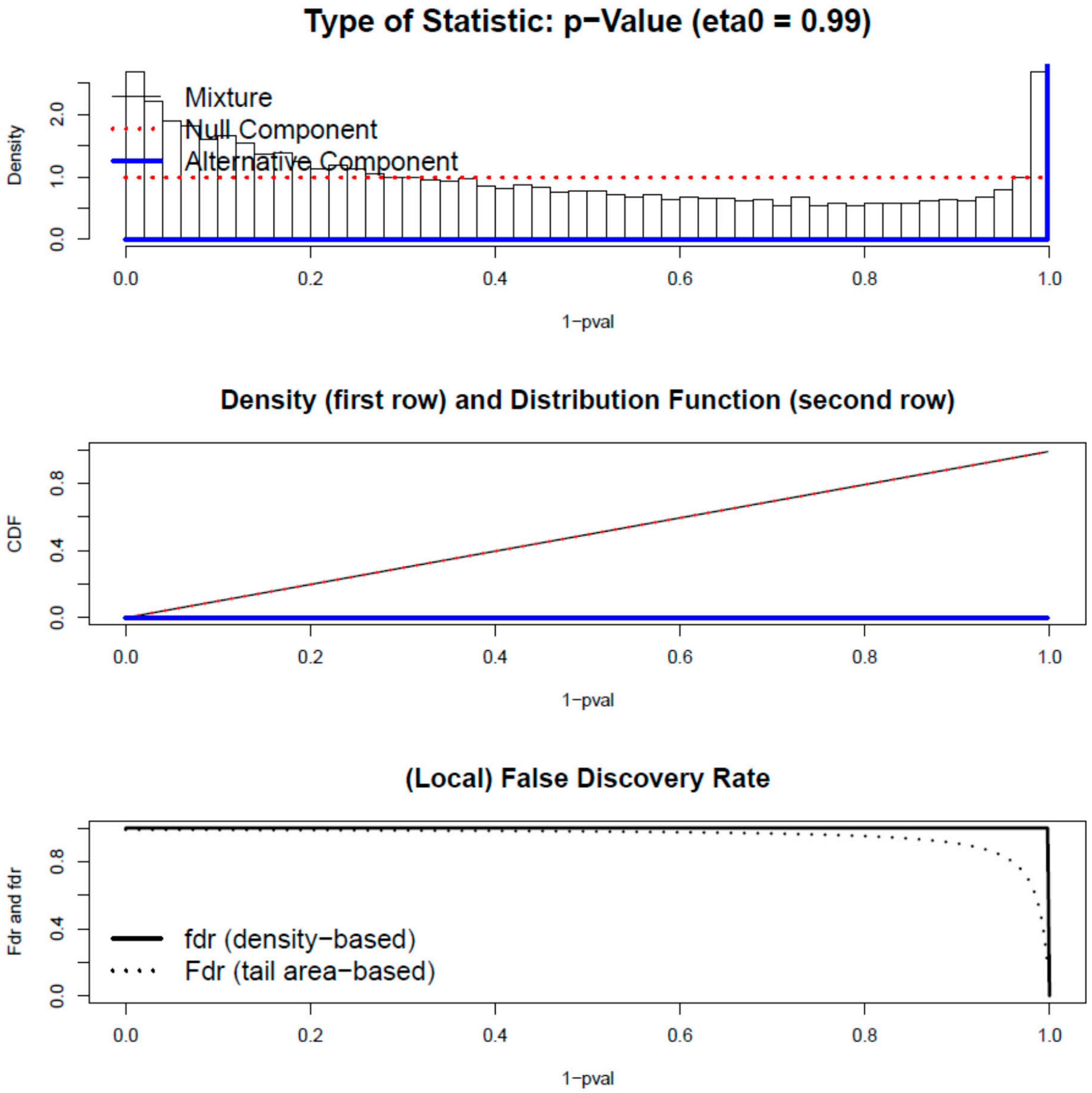

Figure S1. fdrtool's output: mRNA in HCC. **Top**: histogram of 1-*P*; **Middle**: Density and cumulative density of 1-*P*; **Bottom**: (Local) False Discovery Rate.

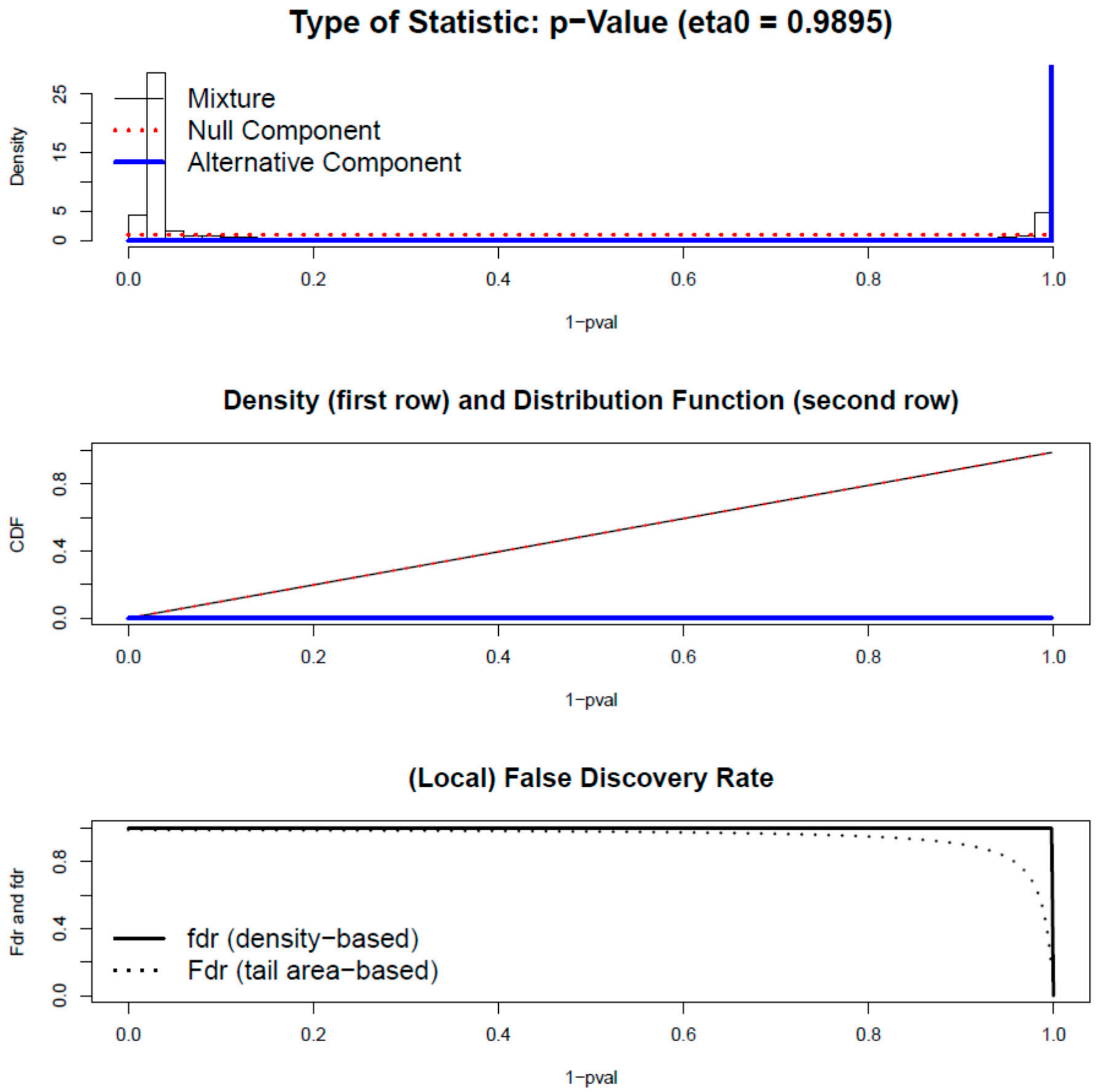

Figure S2. fdrtool's output: miRNA in HCC.

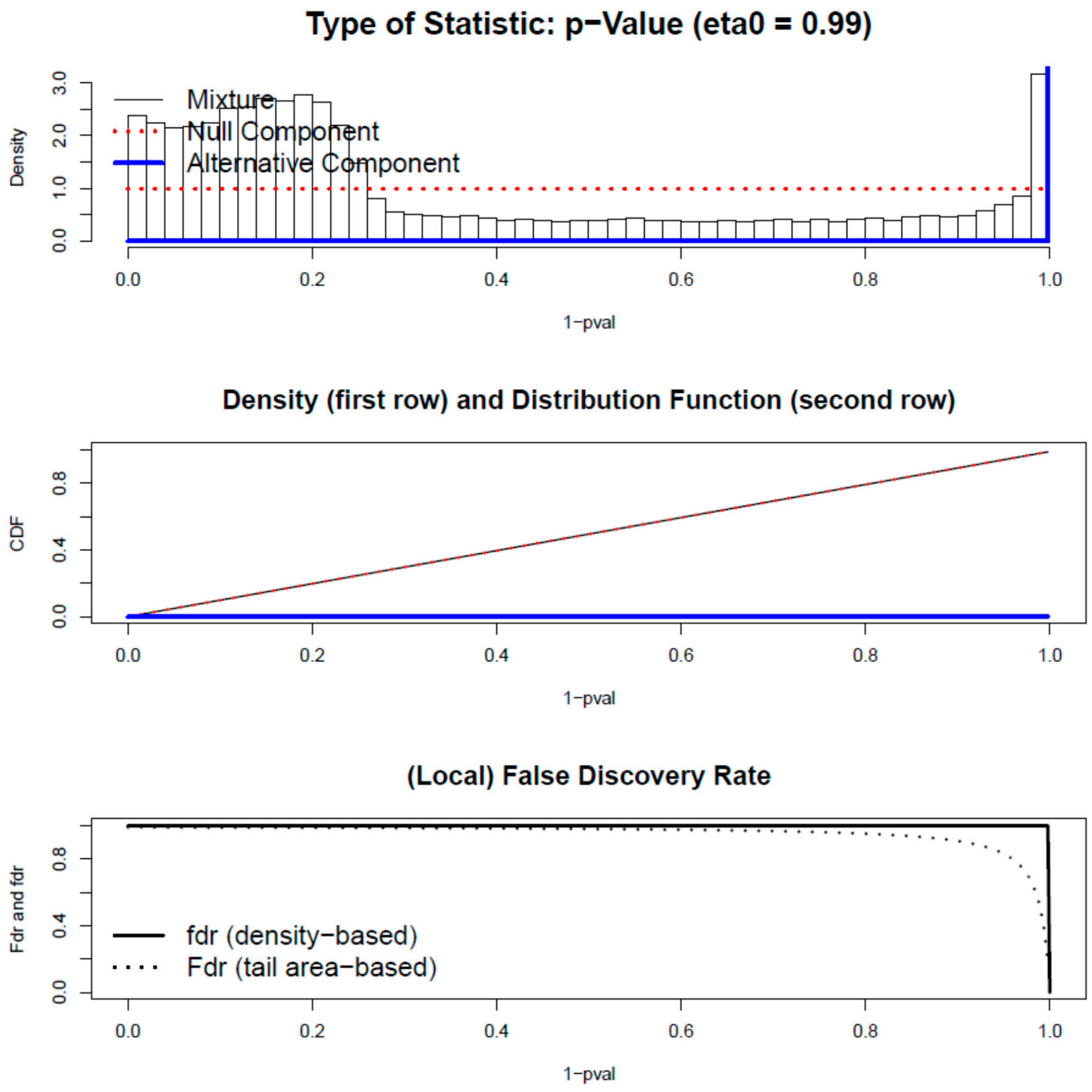

Figure S3. fdrtool's output: mRNA in NSCLC.

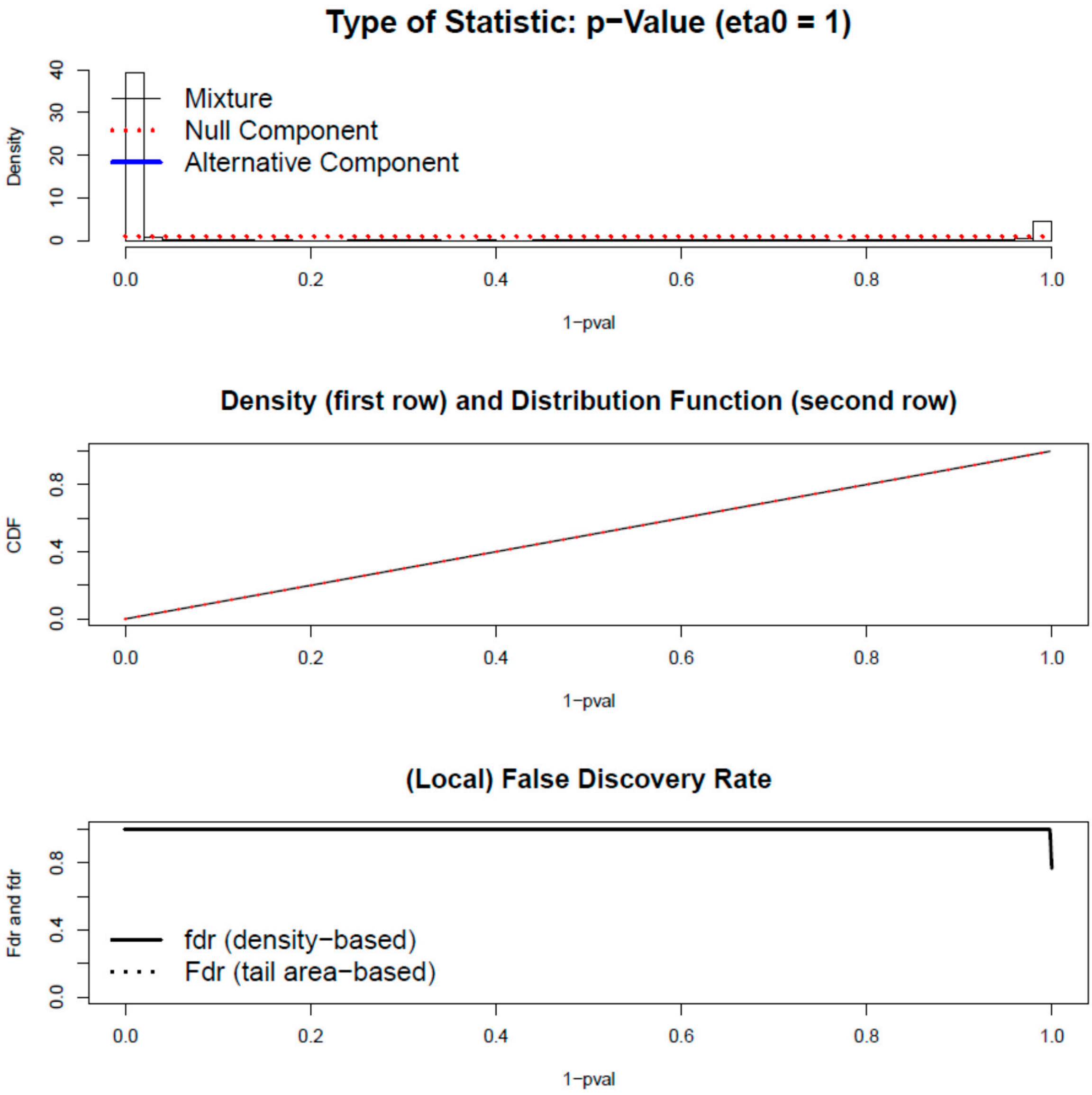

Figure S4. fdrtool's output: miRNA in NSCLC.

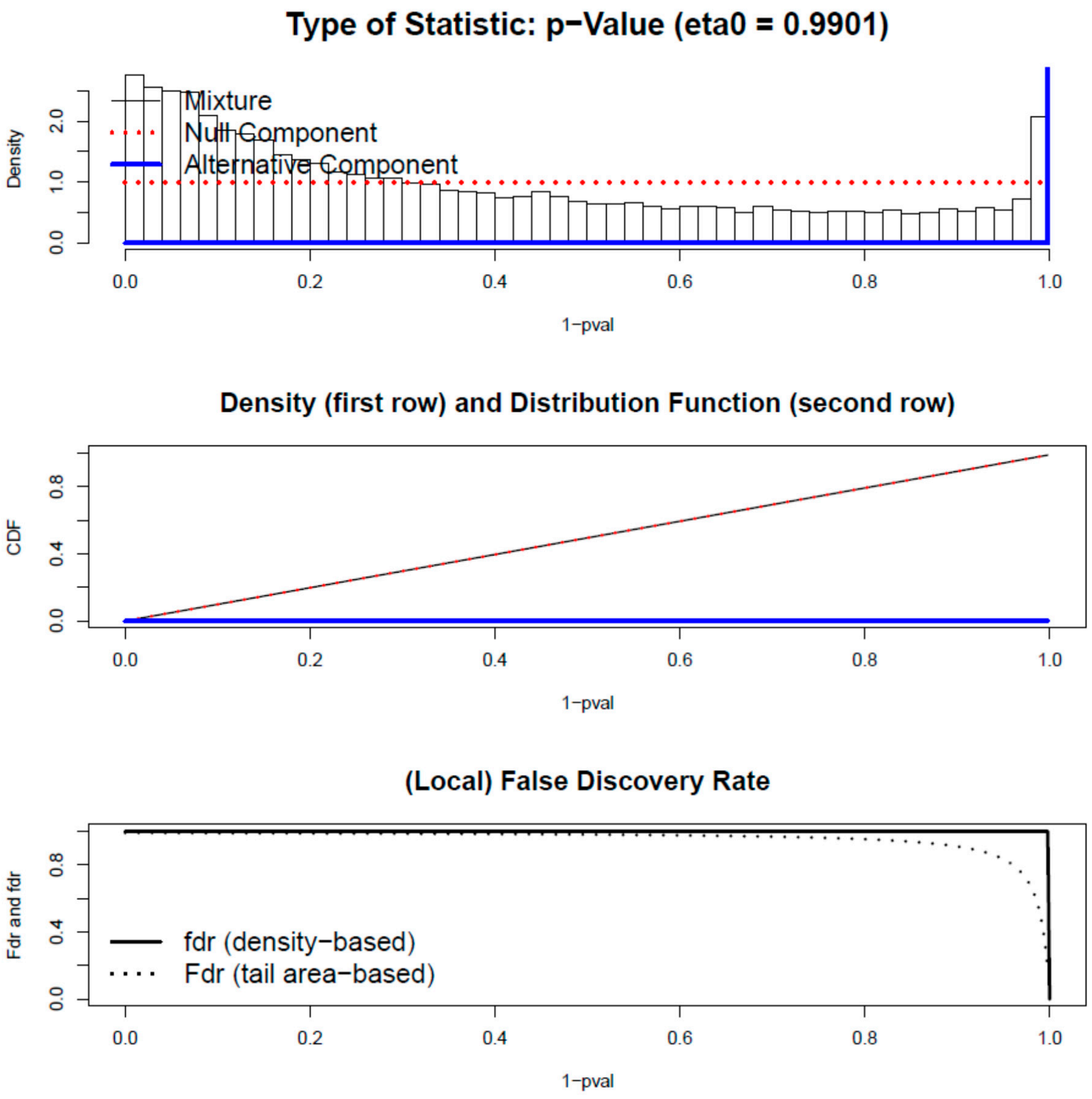

Figure S5. fdrtool's output: mRNA in ESCC.

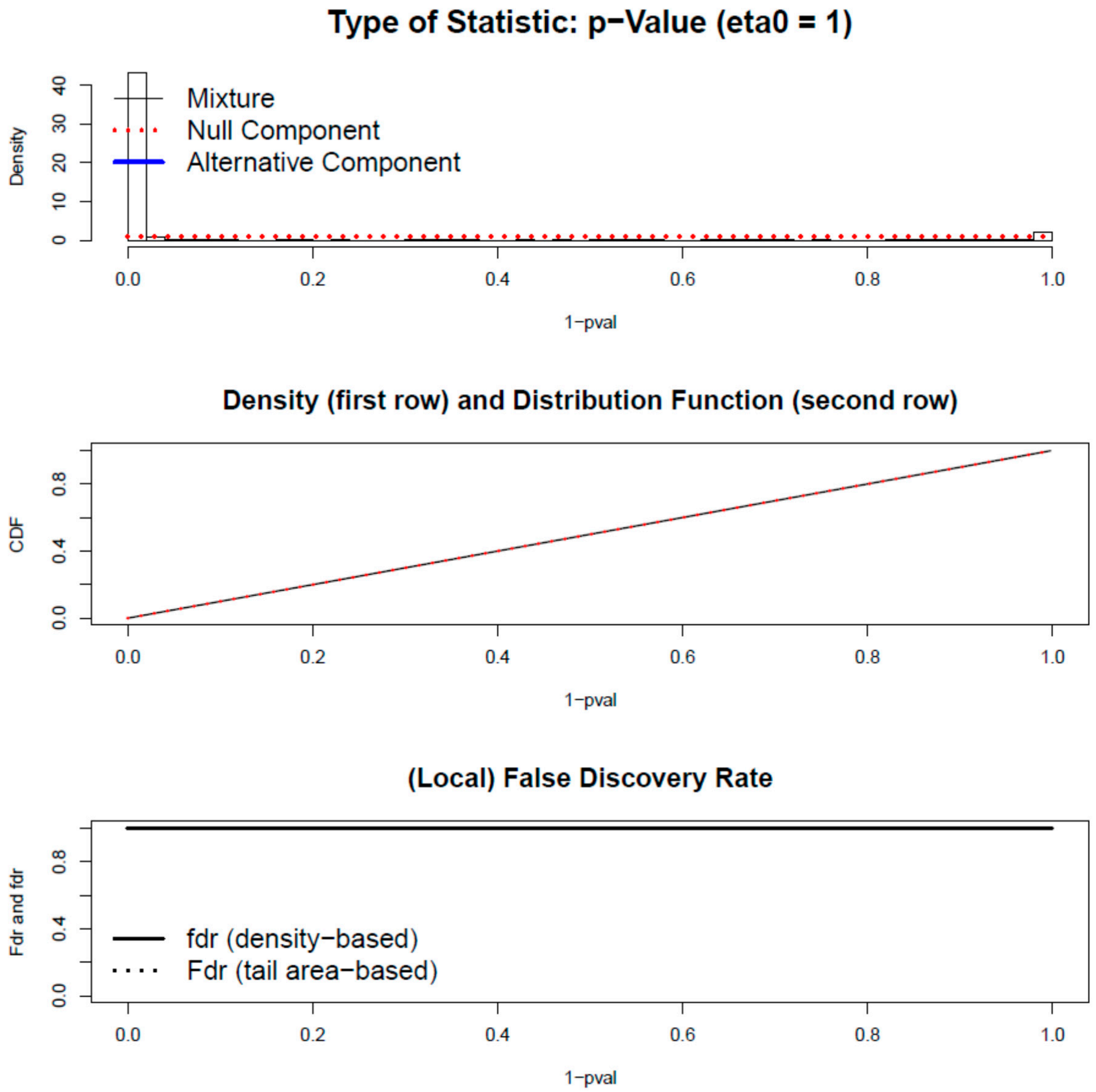

Figure S6. fdrtool's output: miRNA in ESCC.

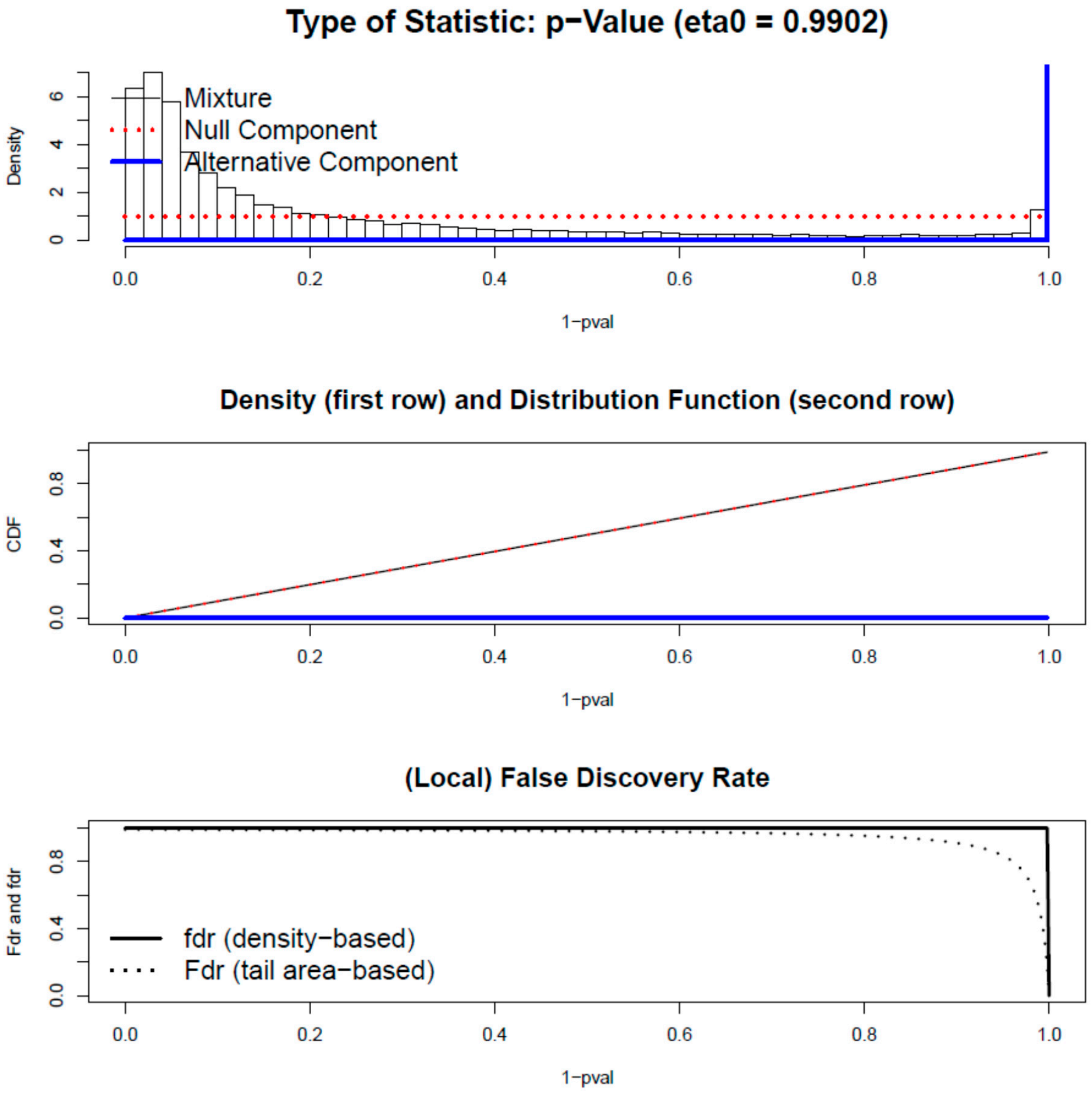

Figure S7. fdrtool's output: mRNA in prostate cancer.

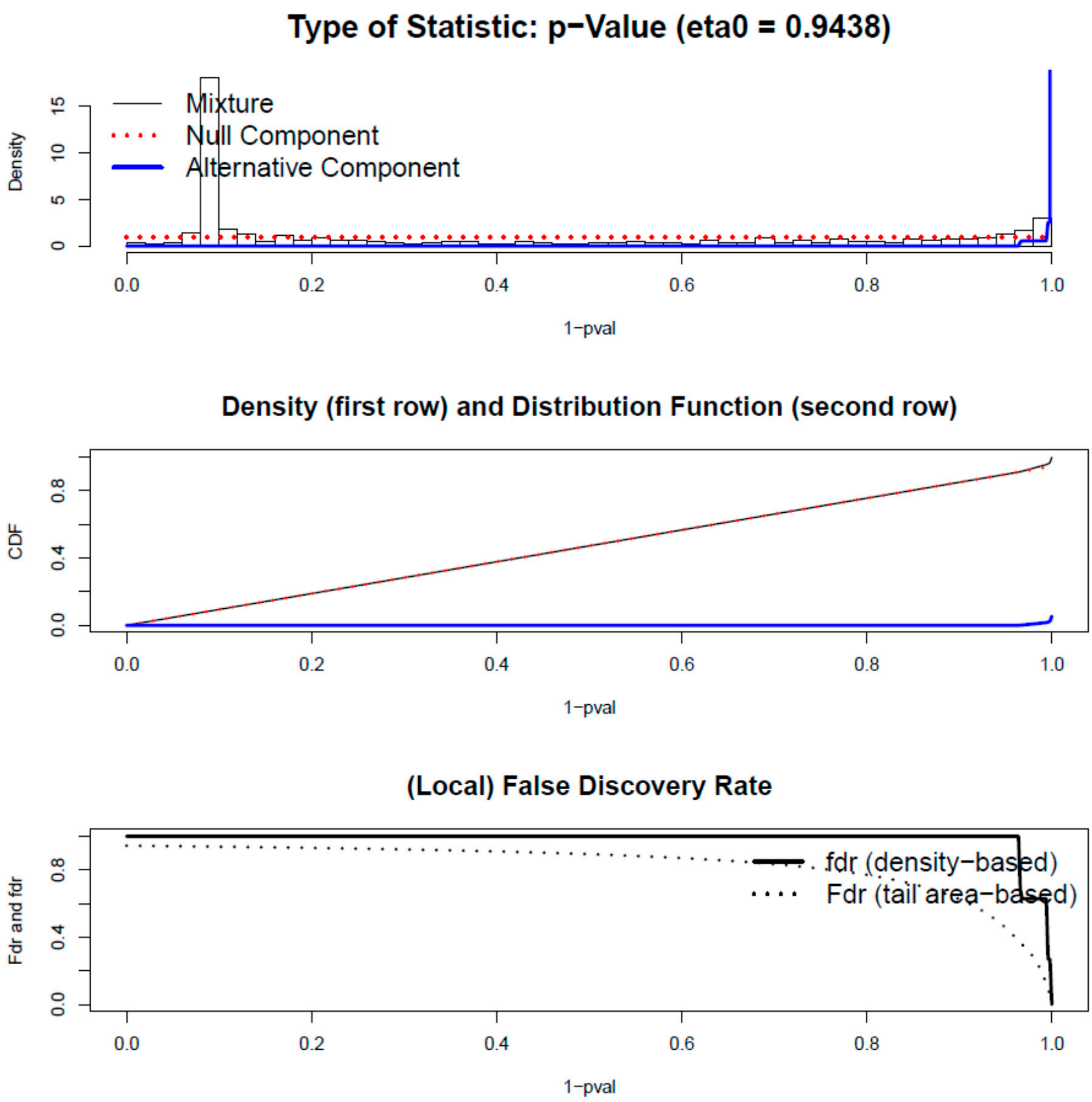

Figure S8. fdrtool's output: miRNA in prostate cancer.

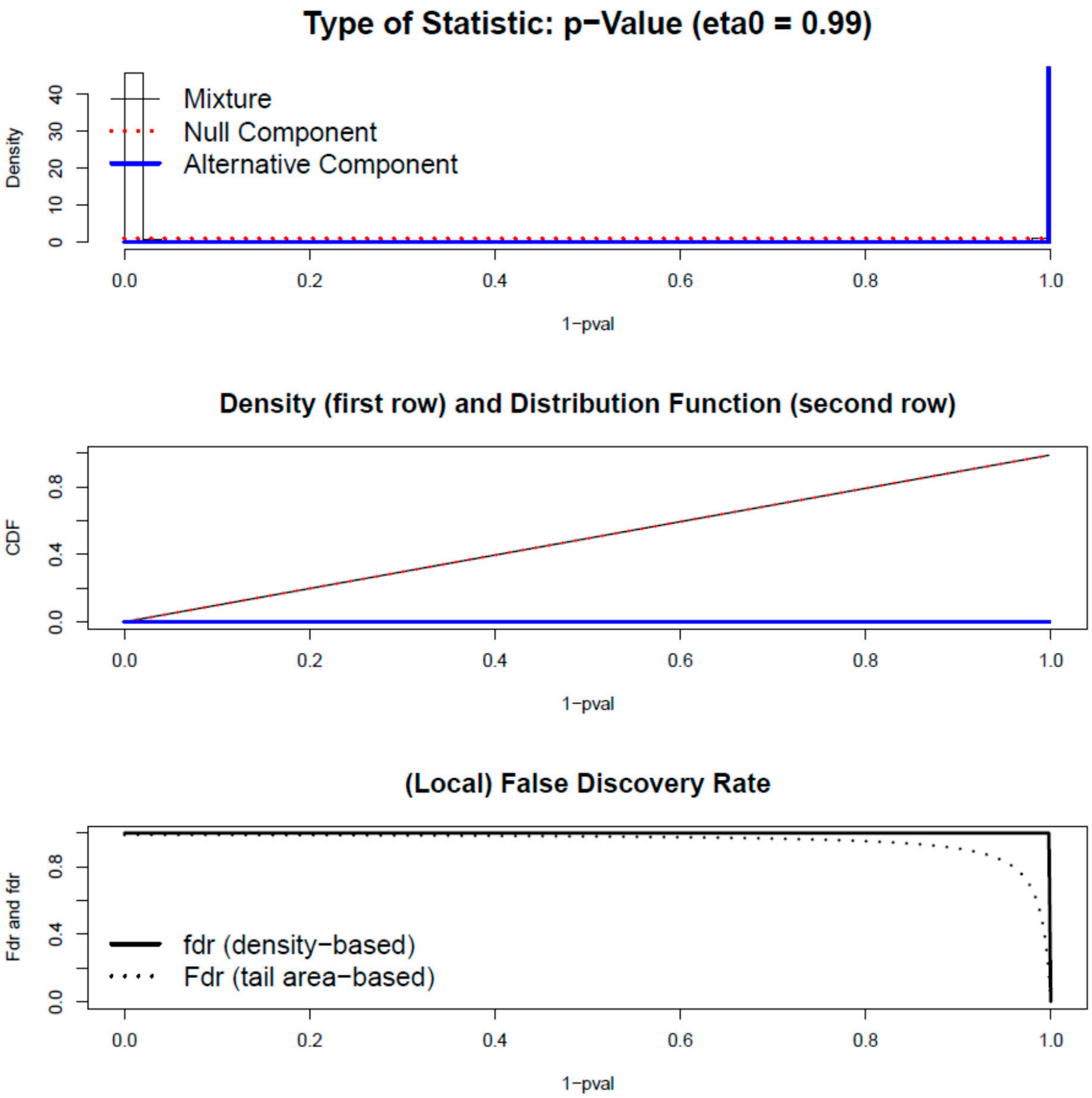

Figure S9. fdrtool's output: mRNA in colon/colorectal cancer.

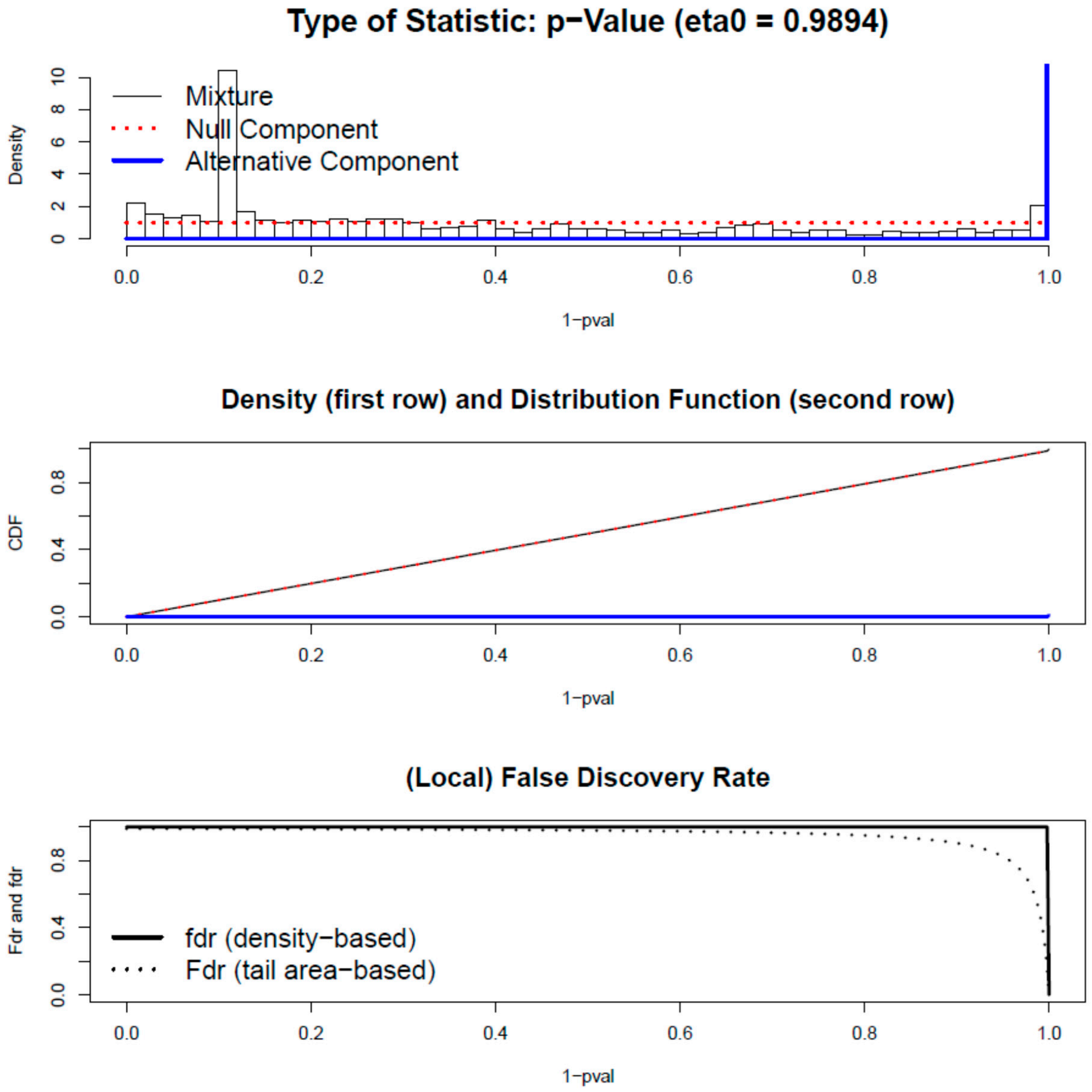

Figure S10. fdrtool's output: miRNA in colon/colorectal cancer.

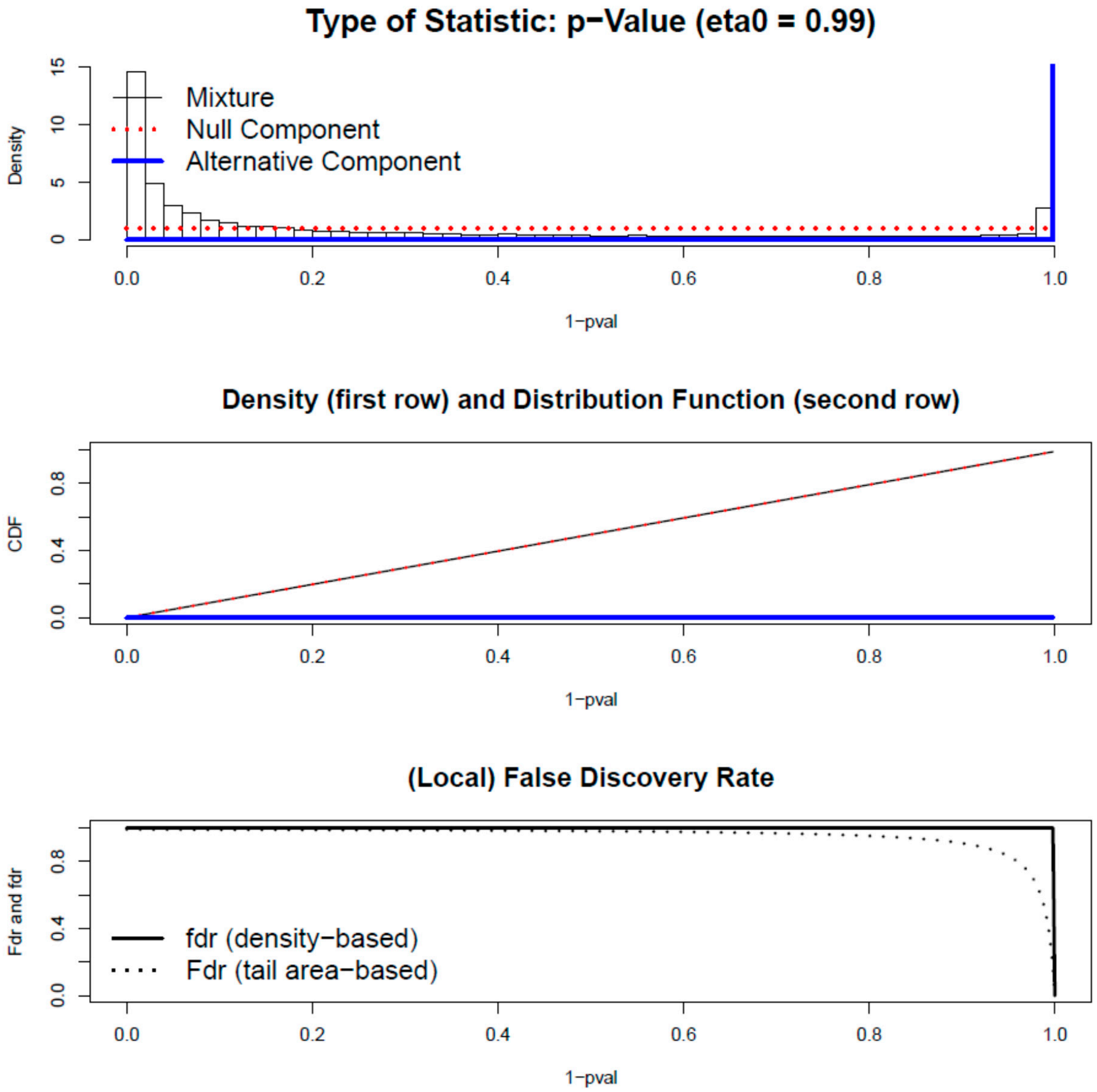

Figure S11. fdrtool's output: mRNA in breast cancer.

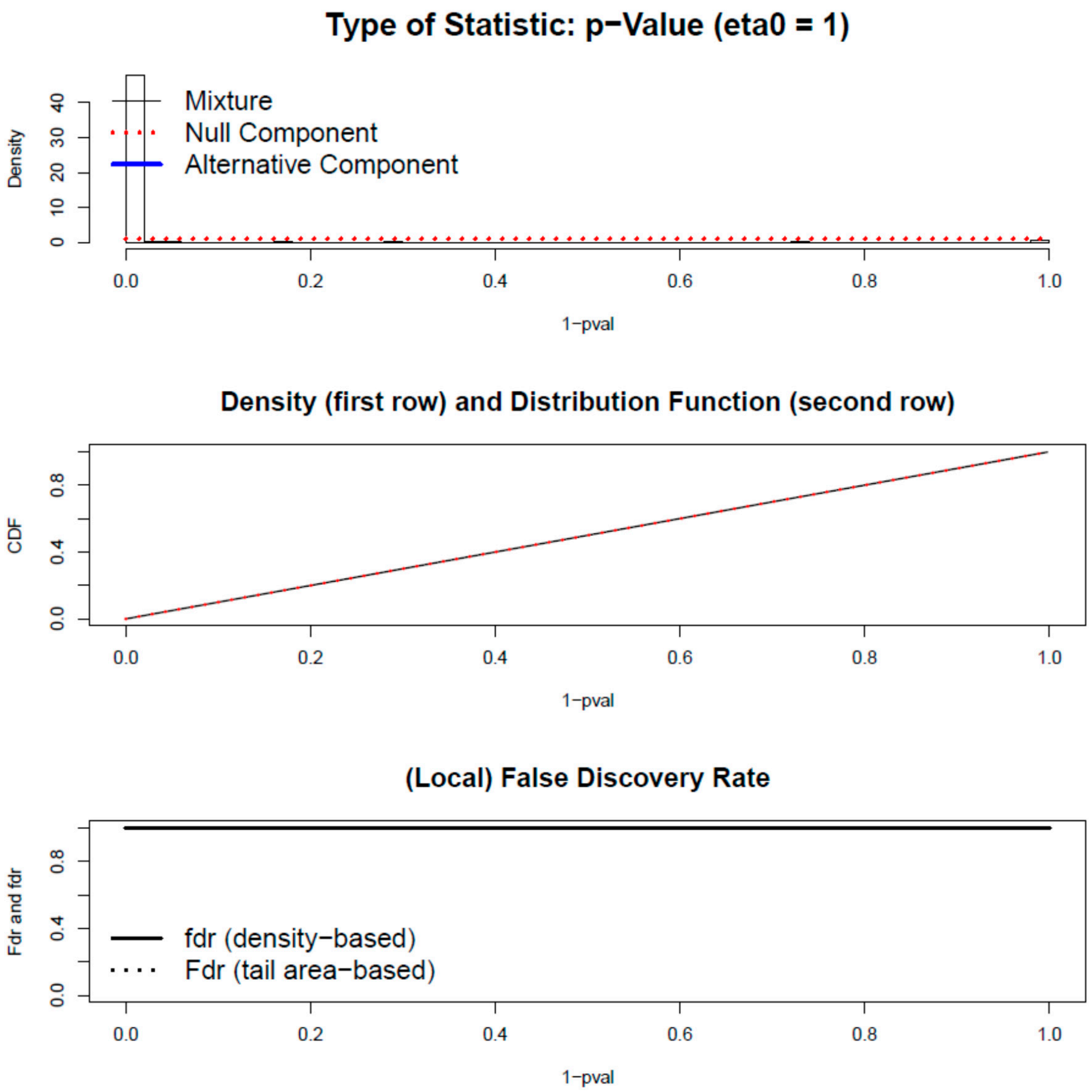

Figure S12. fdrtool's output: miRNA in breast cancer.

Table S1. miRNA–mRNA interaction pairs identified in the HCC samples. $T > N$: upregulated in tumors, $T < N$: upregulated in normal tissues. The starbase column lists the number of cancer cell lines associated with significant reciprocal correlations among the identified pairs. If several miRNAs share the same seed sequence, their numbers are stated separately and connected by a + symbol.

**miRNAs** ($T > N$)	**Gene** ($T < N$)	**Function in HCC**	**Starbase**
miR-93		activates the c-Met/PI3K/Akt pathway [1]	
	ESR1	tumor suppressor [2], promoter methylation [3]	0
	CXCL2	therapeutic target [4], promotes cell proliferation and metastasis [5]	0
miR-25		various roles [6], promotes cell growth [7]	
miR-92a		deregulation [8], contributes to tumor growth [9], preserves sur- vival [10]	
	CD69	CD4(+) CD69(+) CD25(−) T cells were identified in HCC tis- sues [11]	6 + 10
miR-15b		induces endoplasmic reticulum stress and apoptosis [12]	
	CD69	see miR-25/92a	0
	GHR	related to proliferation [13], targets a long non-coding RNA that in-creases EGFR expression [14], distinct therapy treatments between positive/negative tumors [13]	0
	GLS2	tumor suppressor [15], epigenetic silencing [16]	4
miR-148a		repression of HCC cell malignancy [17]	4
	ESR1	see miR-93	
miR-125b		attenuates epithelial-mesenchymal transitions [18]	
	TAT	Loss of one TAT allele [19]	0
let-7a		promising tools against systemic HCC [8]	
	STAB2	upregulation [20], downregulation suppresses tumor [20]	0
	GHR	see miR-15b	0

Table S2. miRNA–mRNA interaction pairs identified in HCC samples (continued).

**miRNAs** ($T < N$)	**Gene** ($T > N$)	**Function in HCC**	**Starbase**
miR-20a		Reduced expression promotes HCC [21]	
	SLC40A1	haplotype [22]	9
	HSPA8	upregulation [23]	4
	GPR88	—	0
	PDE3B	involved in development and metabolism of HCV [24]	2
miR-214		recurrence of HCC [25]	
	PEG10	upregulation [26], associated with poor survival and tumor recurrence [27], promote carcinogenesis [28]	0
miR-195		suppresses angiogenesis and metastasis of HCC [29]	
	GPR88	see miR-20a	0
	GABRE	upregulation [30]	0
miR-200b		suppression of HCC [31]	
	CCNA2	AAV2 integration in CCNA2 [32], associated with low survival sub-class [33]	4
miR-29c		suppression of HCC [34]	
	B3GNT5	—	8
miR-30d		promotes tumor invasion and metastasis [35]	
	B3GNT5	see miR-29c	8

Table S3. miRNA–mRNA interaction pairs identified in the NSCLC samples. *T* > *N*: upregulated in tumors, *T* < *N*: upregulated in normal tissues. For a description of the numbers in the starbase column, see Table S1.

**miRNAs** (*T* < *N*)	**Gene** (*T* > *N*)	**Function in NSCLC**	**Starbase**
miR-30a		inhibits tumor proliferation [36]	
miR-30d		inhibits tumor proliferation [37]	
	BNC1	hypermethylation [38]	0 + 0
	PIGX	candidate driver genes [39]	5 + 4
	GCLC	drug resistance [40]	3 + 2
	THBS2	correlated with decreased vascularity [41]	0 + 0
	HMGB3	overexpression as biomarker [42]	0 + 0
	PFN2	epigenetic regulation of other genes [43]	5 + 4
	FOXD1	association with poor prognosis [44]	4 + 7
	CYP24A1	independent prognostic marker of survival [45]	5 + 5
	PITX1	decreased expression [46]	7 + 7
	BMP7	reported biological impact [47]	0 + 0
	MXRA5	frequent mutation [48]	0 + 0
	SLC7A11	contributes to pathogenesis [49]	6 + 6
	FAP	clinical implications [50]	8 + 11
	BCL11A	overexpression predicts survival and relapse [51]	3 + 5
	ADAM12	diagnostic marker of proliferation, migration and invasion [52]	0 + 0
	ITGA6	downregulation in cancer stem cells [53]	5 + 4
	MARK1	inhibitor, new target agent [54]	0 + 0
	NEFL	candidate biomarker of recurrence and survival [55]	5 + 8
	GRHL1	decreased expression in cell lines [56]	0 + 0
	SLC41A2	—	3 + 6
	GDA	—	1 + 4
	CDCA7	possible subtype-specific expression [57]	9 + 6
	FRMD6	—	6 + 11
	CTHRC1	associated with tumor aggressiveness and poor prognosis [58]	10 + 8
	FBXO32	—	0 + 0
	C3orf58	—	4 + 1
	PPP1R14C	—	2 + 4
	E2F7	upregulated in NSCLC treatment [59]	10 + 5
	SIX1	target of tumor suppressor miRNA [60]	9 + 5
	FAM83F	FAM83B, which belongs to the same protein family as FAM83F, is a novel biomarker [61]	0 + 0

Table S4. miRNA–mRNA interaction pairs identified in NSCLC samples (continued).

**miRNAs** ($T < N$)	**Gene** ($T > N$)	**Function in NSCLC**	**Starbase**
miR-221		Growth inhibitor [62]	
	ATP11B	—	0
	TFAP2A	—	3
	FAT2	subtype-specific biomarker [63]	0
	IGF2BP2	—	0
miR-130a		targets some genes [64]	
	SLC2A1	reported polymorphisms [65]	2
	CALB1	suggested roles [66]	0
	TP63	isoform expression [67]	2
	BCL11A	see miR-30a/30d	1
	SULF1	dysregulation [68]	0
	ADAM12	see miR-30a/30d	3
	CEP55	overexpression and modulation of tumor migration and invasion [69]	3
	GDA	see miR-30a/30d	6
	FRMD6	see miR-30a/30d	4
	E2F7	see miR-30a/30d	2
miR-100		tumor suppressor [70]	
	FGFR3	mutation [71]	6
	GRHL1	miR-30a/30d	7
miR-223		potent tumor suppressor [72]	
	PFN2	miR-30a/30d	0
	ECT2	overexpression [73]	3
miR-143		inhibits NSCLC cell growth and metastasis [74]	
	COL1A1	overexpression [75]	0
	COL5A1	—	0
	PLAU	subtype-specific expression [76]	0
	KLF5	inhibits apoptosis [77]	8
	SLC7A11	chemoresistance [78]	4
	ITGA6	miR-30a/30d	2
	GOLM1	overexpression [79]	5
	COL5A2	overexpression [80]	0
	FAM83F	see miR-30a/30d	0

Table S5. miRNA–mRNA interaction pairs identified in NSCLC samples (continued).

miRNAs (*T* < *N*)	Gene (*T* > *N*)	Function in NSCLC	Starbase
miR-145		inhibits NSCLE proliferation [81]	
	ABCC1	drug resistance [82]	3
	HMGB3	miR-30a/30d	6
	TFRC	frequent amplification [83]	7
	KLF5	miR-143	4
	SULF1	see miR-130a	0
	PSAT1	sustains proliferation [84]	6
miR-125b		downregulation promotes NSCLC invasion and migration [85]	
	HMGB3	miR-30a/30d	7
	MMP11	correlated with higher-grade NSCLC [86]	0
	CYP24A1	miR-30a/30d	0
	RAPGEFL1	—	0
	GRHL1	miR-30a/30d	5
	VANGL2	—	0
	FAM83F	see miR-30a/30d	
miR-195		tumor suppressor [87]	
	ANLN	plays a critical role in NSCLC [88]	7
	AK4	promotes metastasis [88]	0
	KCNN4	associated with poor prognosis [89]	1
	CXCL10	blood biomarker [90]	1
	KIF23	overexpression [91]	8
	PTHLH	increased in the serum and urine of NSCLE patients [92]	3
	HMGA2	overexpression [93]	8
	LRRC15	—	0
	RAPGEFL1	see miR-125b	0
	FZD10	overexpression [94]	1
	PLUNC	biomarker [95]	0
	RGMA	—	0
	SLC41A2	see miR-30a/30d	2
	KCTD1	—	5
	FAM110C	—	3
	E2F7	see miR-30a/30d	8
	PRR11	cell cycle progression [96]	0
	ODZ2	biomarker [97]	0

Table S6. miRNA–mRNA interaction pairs identified in NSCLC samples (continued).

**miRNAs** ($T < N$)	**Gene** ($T > N$)	**Function in NSCLC**	**Starbase**
miR-26a		drug resistance [98]	
	COL1A2	potential therapeutic target [99]	0
	PLOD2	prognostic variables [100]	8
	ADM	regulates NSCLC [101]	7
	LOXL2	downregulation [102]	10
	COL5A1	see miR-143	8
	COL11A1	overexpression [103]	0
	TFAP2A	see miR-221	5
	COL10A1	overexpression [104]	0
	WNT5A	promotes angiogenesis [105]	2
	CILP	—	0
	HMGA2	see miR-195	6
	JAG1	prognostic biomarkers [106]	4
	TP63	see miR-130a	0
	SLC7A11	see miR-143	6
	BCL11A	see miR-30a/30d	2
	SULF1	see miR-130a	0
	ADAM12	see miR-30a/30d	0
	ITGA6	see miR-143	5
	MARK1	see miR-30a/30d	3
	HAS3	abundant expression [107]	0
	VANGL2	see miR-125b	1
	E2F7	miR-30a/30d	8
	LPAR3	—	4
	GRHL3	—	0

Table S7. miRNA–mRNA interaction pairs identified in NSCLC samples (continued).

**miRNAs** ($T < N$)	**Gene** ($T > N$)	**Function in NSCLC**	**Starbase**
miR-29a		aberrant methylation [108]	
	COL1A1	see miR-143	4
	COL3A1	upregulation [109]	4
	SLC16A1	—	7
	COL1A2	see miR-26a	0
	LOXL2	see miR-26a	6
	COL5A1	see miR-26a	4
	COL11A1	see miR-26a	0
	DSG3	biomarker [110]	0
	PTHLH	see miR-195	5
	UPK1B	a part of classifier [111]	0
	BCL11A	see miR-30a/30d	4
	ADAM12	see miR-30a/30d	6
	COL4A6	frequently mutated=[48]	0
	ITGA6	see miR-30a/30d	6
	RAPGEFL1	see miR-125b	0
	COL5A2	see miR-143	6
	HAS3	see miR-26a	3
	KCTD1	see miR-195	6
	E2F7	see miR-30a/30d	9

Table S8. miRNA–mRNA interaction pairs identified in NSCLC samples (continued).

miRNAs (*T* > *N*)	Gene (*T* < *N*)	Function in NSCLC	Starbase
miR-106a		promotes growth and metastasis of NSCLC [112]	
miR-17		cancer development [113]	
	IGSF10	differently expressed [114]	0 + 0
	SLC4A4	—	0 + 0
	LMO3	cell growth and metastasis [115]	0 + 0
	NR4A2	tumor suppressor [116]	5 + 6
	HLF	—	0 + 0
	FOXF1	drives NSCLC progression [117]	8 + 9
	SMAD6	contributes to patient survival [118]	4 + 8
	CD69	detected expression [119]	6 + 9
	DLC1	tumor suppressor [120]	9 + 9
	TMEM100	downregulation [114]	9 + 8
	TBX3	overexpression [121]	5 + 6
	COL4A3	correlated with poor prognosis [122]	0 + 0
	NTN4	—	6 + 10
	SCD5	abundance [123]	4 + 5
	TMTC1	—	6 + 9
	PTPN21	mutations [124]	9 + 8
	NEDD4L	prognostic marker [125]	3 + 4
	ANKRD29	—	9 + 11
	GLDN	—	0 + 0
	ATP11A	—	6 + 3
	GPR133	mutation [126]	7 + 10
	NCKAP5	downregulation [114]	4 + 6

Table S9. miRNA–mRNA interaction pairs identified in NSCLC samples (continued).

miRNAs (*T* > *N*)	Gene (*T* < *N*)	Function in NSCLC	Starbase
miR-141		biomarker [127]	
miR-200a		targets multiple NSCLC prognostic markers [128]	
	PLCB4	downregulation [129]	0 + 0
	LMO3	see miR-106a/17	5 + 5
	HLF	see miR-106a/17	0 + 0
	GPM6B	—	0 + 0
	PDZD2	—	0 + 0
	PTCH1	suppressed by miR-212 [130]	4 + 0
	GATA6	tumor inhibitor [131]	6 + 5
	FOXA2	downregulated [132]	0 + 0
	DLC1	see miR-106a/17	11 + 9
	ATP8A1	downregulated [133]	5 + 5
	CLIC5	downregulated [134]	0 + 0
	SLC1A1	polymorphisms [65]	4 + 3
	PCDH9	possible association with NSCLC development, metastasis and prognosis [135]	11 + 8
	SOX17	promoter of methylation in plasma-circulating tumors DNA [136]	0 + 0
	SCD5	see miR-106a/17	5 + 4
	SEMA6A	downregulation [137]	0 + 0
	HSPC159	—	0 + 0
	TMTC1	see miR-106a/17	0 + 0
	SYNPO2	upregulated under HDACi treatment [138]	0 + 0
	SCN7A	associated with good survival [139]	0 + 0
	ADRB1	ADRB1-specific or non-selective drug improves survival [140]	4 + 2
	NCKAP5	see miR-106a/17	3 + 2
	PAQR5	—	2 + 3

Table S10. miRNA–mRNA interaction pairs identified in NSCLC samples (continued).

miRNAs (*T* >*N*)	Gene (*T* <*N*)	Function in NSCLC	Starbase
miR-200b		targets multiple NSCLC prognostic markers [128]	
miR-200c		inhibits invasion and metastasis [141]	
	DUSP1	promotes angiogenesis, invasion and metastasis [142]	7 + 10
	FHL1	downregulation [143]	10 + 9
	SLC4A4	see miR-106a/17	0 + 0
	REEP1	upregulated under both 5-dAzaC treatment and BRG1 re-expression [144]	4 + 5
	HLF	see miR-106a/17	0 + 0
	DACH1	tumor suppressor [145]	0 + 1
	FOXF1	see miR-106a/17	9 + 8
	GPM6A	differential expression [146]	7 + 8
	CHRDL1	aberrant gene expression [147]	0 + 0
	PTCH1	see miR-141/200a	2 + 3
	DLC1	see miR-106a/17	8 + 9
	KLF4	metastasis regulation [148]	4 + 2
	COL4A3	see miR-106a/17	6 + 6
	PTPN21	see miR-106a/17	7 + 8
	SEMA6D	—	5 + 3
	NEDD4L	see miR-106a/17	1 + 1
	AFF3	—	0 + 0
	GCOM1	downregulation [149]	3 + 5
	MAMDC2	aberrant methylation [150]	7 + 10
	PAQR5	see miR-141/200a	0 + 0

Table S11. miRNA–mRNA interaction pairs identified in NSCLC samples (continued).

miRNAs (*T* > *N*)	Gene (*T* <*N*)	Function in NSCLC	Starbase
miR-205		drives malignant phenotype [151]	
	LPCAT1	upregulation [152]	4
	SLC4A4	see miR-106a/17	0
	FOXF1	see miR-106a/17	7
	TBX3	see miR-106a/17	0
	SCD5	see miR-106a/17	0
	AFF3	see miR-200b/200c	0
	GCOM1	see miR-200b/200c	0
	LRRK2	—	0
miR-21		restrains cell proliferation and migration [153]	
	THBD	can be used as a part of survival prediction [154]	0
	PDZD2	see miR-141/200a	8
	CD69	see miR-106a/17	0
	PTCH1	see miR-141/200a	0
	OLR1	can be used as a part of prognosis prediction [155]	2
	LIFR	—	11
	ARHGEF26	—	0
	SOX7	downregulation [156]	7
	KCNT2	—	0

Table S12. miRNA–mRNA interaction pairs identified in NSCLC samples (continued).

**miRNAs** (*T* > *N*)	**Gene** (*T* < *N*)	**Function in NSCLC**	**Starbase**
miR-19b		circulating biomarker [157]	
	TGM2	cisplatin resistance marker [158]	9
	EDNRB	Aberrant promoter methylation [159]	8
	REEP1	see miR-200b/200c	3
	HLF	see miR-106a/17	0
	DLC1	see miR-106a/17	8
	LIMCH1	inclusion in tumorgenesis [160]	9
	PARM1	—	8
	CAB39L	—	0
	FRAS1	knockdown reduces A549 cell migration and invasion [161]	3
	HSPC159	see miR-141/200a	0
	PTPN21	see miR-106a/17	9
	VSIG10	—	2
	SEMA6D	see miR-200b/200c	4
	ARHGEF26	see miR-21	0
	SYNPO2	see miR-141/200a	0
	ADRB1	see miR-141/200a	9
	LRRK2	see miR-205	0
	ATP11A	see miR-106a/17	2
	SCN4B	—	0
	NCKAP5	see miR-106a/17	6
	KCNT2	see miR-21	0

Table S13. miRNA–mRNA interaction pairs identified in ESCC samples. *T* > *N*: upregulated in tumors, *T* < *N*: upregulated in normal tissues. For a description of the numbers in the starbase column, see Table S1.

**miRNAs** (*T* < *N*)	**Gene** (*T* > *N*)	**Function in ESCC**	**Starbase**
miR-203		G1 arrest [162],downregulation [163]	
	BIRC5	overexpression [164]	1
	TP63	tumor suppressor [165]	0
(*T* > *N*	(*T* < *N*)		
miR-223		clinically significant [166]; overexpression regulates the ubiquitin ligase FBXW7 [167]	
	HLF	lineage - specifically essential for tumor growth [168]	0
	SORBS1	significantly shortened tandem 3′ UTRs [169]	6

Table S14. miRNA–mRNA interaction pairs identified in prostate cancer samples. *T* > *N*: upregulated in tumors, *T* < *N*: upregulated in normal tissues. For a description of the numbers in the starbase column, see Table S1.

**miRNAs** (*T* > *N*)	**Gene** (*T* < *N*)	**Function in Prostate Cancer**	**Starbase**
miR-136		biomarker [170]	
	EIF4B	related to anticancer activity [171]	6
	HOXC10	aberrant expression [172]	0
miR-145		suppress the androgen receptor [173]	
	TFRC	biomarker [174]	7
	EIF4B	see miR-136	0
	KIAA0355	—	0
	USP31	—	2
	ZDHHC8	—	0
miR-22		overexpression in DU145 [175]	
	KIAA0355	see miR-145	7
	PTEN	genomic rearrangement [176]	2
	ZNF827	—	0
miR-494		suppress proliferation, invasion, and migration [177]	
	C14orf43	—	2
	YEATS2	—	1
	PTEN	see miR-22	1
	USP31	see miR-145	1
	USP27X	—	0

Table S15. miRNA–mRNA interaction pairs identified in prostate cancer samples (continued).

**miRNAs** (*T* < *N*)	**Gene** (*T* > *N*)	**Function in Prostate Cancer**	**Starbase**
let-7a/7f		inclusion in tumorgenesis [178]	
	NXT2	—	0 + 1
	ANKRD12	—	1 + 2
	FOXP1	tumor suppressor [179]	3 + 1
	RTCD1	—	7 + 5
	PLEKHG6	—	0 + 0
miR-200a		tumor suppressor [180]	
	FOXP1	see let-7a/7f	6
miR-200b		tumor suppressor [180]	
	FOXP1	see let-7a/7f	0
	CADM1	inactivated by methylation promoter [181]	2
	PFN2	—	0
miR-20b		reported importance [182]	
	ANKRD12	see let-7a/7f	3
	RGNEF	—	3
	PFN2	see miR-200b	1

Table S16. miRNA–mRNA interaction pairs identified in colorectal/colon cancer samples. *T* > *N*: upregulated in tumors, *T* < *N*: upregulated in normal tissues. For a description of the numbers in the starbase column, see Table S1.

**miRNAs** (*T* < *N*)	**Gene** (*T* > *N*)	**Function in Colorectal/Colon Cancer**	**Starbase**
miR-182		deregulation promotes proliferation [183]	
	FN1	upregulation [184]	0
miR-183		overexpression [185]	
	EEF2	knockdown inhibits growth [186]	4
miR-96		overexpression [187]	
	FN1	see miR-182	9
(*T* > *N*)	(*T* < *N*)		
miR-133a		tumor suppressor [188]	
	PTPRO	sensitization [189]	0
miR-137		tumor suppressor [190]	
	SLC25A5	—	3
miR-149		tumor suppressor [191]	
	IGJ	downregulation [192]	0
miR-30a		targets the insulin receptor [193]	
	MYH11	down-regulated expression correlates with poor prognosis [194]	0
	CEACAM1	regulates metastasis [195]	0

Table S17. miRNA–mRNA interaction pairs identified in breast cancer samples. *T* > *N*: upregulated in tumors, *T* < *N*: upregulated in normal tissues. For a description of the numbers in the starbase column, see Table S1.

**miRNAs** (*T* > *N*)	**Gene** (*T* < *N*)	**Function in Breast Cancer**	**Starbase**
let-7i		regulates self-renewal and tumorigenicity [196]	
	AMT	subtype-specific downregulation [197]	4
	GHR	up-expression [198]	0
	HOXA9	modulates tumor phenotype [199]	0
miR-148a		inhibits migration of breast cancer cells [200]	
	CNN1	possible therapeutic target [201]	0
	FOSB	overexpression [202]	0
	TNXB	adhesion modulation [203]	0
miR-19b		regulates tissue factor expression [204]	
	NCALD	frequent amplification [205]	0
	HOXA9	see let-7i	1
	IRX4	—	0
	CXCL12	promotes metastasis [206]	0
	MATN2	downregulation [207]	9
miR-21		overexpression [208]	
	MSX1	—	6
	MATN2	see miR-19b	9
miR-23a		overexpression [209]	
	SFRP1	associated with neoadjuvant chemotherapy [210]	0
	FOSB	see miR-148a	0
	CXCL12	see miR-19b	5
miR-24		enhances tumor invasion and metastasis [211]	
	NCALD	see miR-19b	0

Table S18. miRNA–mRNA interaction pairs identified in breast cancer samples (continued).

miRNAs (*T* < *N*)	Gene (*T* > *N*)	Function in Breast Cancer	Starbase
let-7b		regulates self-renewal and tumorigenicity [196]	
	ICOS	associated with poor prognosis [212]	0
	SYT1	possible biomarker [213]	0
	ERO1L	associated with overall good survival [214]	4
	CTSC	metastasis [215]	3
	S100A8	aggression [216]	0
	RDH10	—	4
miR-125b		targets ARID3B [217]	
	DUSP6	silencing inhibits proliferation [218]	5
	CCR2	coordinates survival and motility [219]	0
	BIN2	also known as breast cancer-associated protein 1 [220]	0
	WARS	good prognostic marker [221]	5
	PDE7A	—	4
	GALNT7	—	5
	GLS	promotes proliferation [222]	0
miR-143		tumor suppressor [223]	
	WHSC1	positive regulator of ERα signaling [224]	0
	ITGA6	overexpression [225]	2
miR-145		tumor suppressor [223]	
	DUSP6	see miR-125b	1
	RPS6KB1	alteration [226]	3
	ITPR2	fused with ETV6 [227]	0
miR-22		biomarker [228]	
	FOSL1	overexpression inhibition decreases cell growth [229]	2

References

1. Ohta, K.; Hoshino, H.; Wang, J.; Ono, S.; Iida, Y.; Hata, K.; Huang, S.K.; Colquhoun, S.; Hoon, D.S. MicroRNA-93 activates c-Met/PI3K/Akt pathway activity in hepatocellular carcinoma by directly inhibiting PTEN and CDKN1A. *Oncotarget* **2015**, *6*, 3211–3224.
2. Hishida, M.; Nomoto, S.; Inokawa, Y.; Hayashi, M.; Kanda, M.; Okamura, Y.; Nishikawa, Y.; Tanaka, C.; Kobayashi, D.; Yamada, S.; *et al*. Estrogen receptor 1 gene as a tumor suppressor gene in hepatocellular carcinoma detected by triple-combination array analysis. *Int. J. Oncol.* **2013**, *43*, 88–94.
3. Dou, C.Y.; Fan, Y.C.; Cao, C.J.; Yang, Y.; Wang, K. Sera DNA Methylation of CDH1, DNMT3b and ESR1 Promoters as Biomarker for the Early Diagnosis of Hepatitis B Virus-Related Hepatocellular Carcinoma. *Dig. Dis. Sci.* **2015**, doi:10.1007/s10620-015-3975-3.
4. Wilson, C.L.; Jurk, D.; Fullard, N.; Banks, P.; Page, A.; Luli, S.; Elsharkawy, A.M.; Gieling, R.G.; Chakraborty, J.B.; Fox, C.; *et al*. NFκB1 is a suppressor of neutrophil-driven hepatocellular carcinoma. *Nat. Commun.* **2015**, *6*, 6818.
5. Song, X.; Wang, Z.; Jin, Y.; Wang, Y.; Duan, W. Loss of miR-532-5p in vitro promotes cell proliferation and metastasis by influencing CXCL2 expression in HCC. *Am. J. Transl. Res.* **2015**, *7*, 2254–2261.
6. Li, Y.; Tan, W.; Neo, T.W.; Aung, M.O.; Wasser, S.; Lim, S.G.; Tan, T.M. Role of the miR-106b-25 microRNA cluster in hepatocellular carcinoma. *Cancer Sci.* **2009**, *100*, 1234–1242.
7. Wang, C.; Wang, X.; Su, Z.; Fei, H.; Liu, X.; Pan, Q. miR-25 promotes hepatocellular carcinoma cell growth, migration and invasion by inhibiting RhoGDI1. *Oncotarget* **2015**, *6*, 36231–36244.
8. Shigoka, M.; Tsuchida, A.; Matsudo, T.; Nagakawa, Y.; Saito, H.; Suzuki, Y.; Aoki, T.; Murakami, Y.; Toyoda, H.; Kumada, T.; *et al*. Deregulation of miR-92a expression is implicated in hepatocellular carcinoma development. *Pathol. Int.* **2010**, *60*, 351–357.
9. Yang, W.; Dou, C.; Wang, Y.; Jia, Y.; Li, C.; Zheng, X.; Tu, K. MicroRNA-92a contributes to tumor growth of human hepatocellular carcinoma by targeting FBXW7. *Oncol. Rep.* **2015**, *34*, 2576–2584.
10. Urtasun, R.; Elizalde, M.; Azkona, M.; Latasa, M.U.; Garcia-Irigoyen, O.; Uriarte, I.; Barrena, M.G.F.; Vicent, S.; Alonso, M.M.; Muntane, J.; *et al*. Splicing regulator SLU7 preserves survival of hepatocellular carcinoma cells and other solid tumors via oncogenic miR-17-92 cluster expression. *Oncogene* **2016**, doi:10.1038/onc.2015.517.

11. Zhu, J.; Feng, A.; Sun, J.; Jiang, Z.; Zhang, G.; Wang, K.; Hu, S.; Qu, X. Increased CD4(+) CD69(+) CD25(−) T cells in patients with hepatocellular carcinoma are associated with tumor progression. *J. Gastroenterol. Hepatol.* **2011**, *26*, 1519–1526.
12. Yang, Y.; Hou, N.; Wang, X.; Wang, L.; Chang, S.; He, K.; Zhao, Z.; Zhao, X.; Song, T.; Huang, C. miR-15b-5p induces endoplasmic reticulum stress and apoptosis in human hepatocellular carcinoma, both *in vitro* and *in vivo*, by suppressing Rab1A. *Oncotarget* **2015**, *6*, 16227–16238.
13. Li, S.; Hou, G.; Wang, Y.; Su, X.; Xue, L. Influence of recombinant human growth hormone (rhGH) on proliferation of hepatocellular carcinoma cells with positive and negative growth hormone receptors *in vitro*. *Tumori* **2010**, *96*, 282–288.
14. Qi, H.L.; Li, C.S.; Qian, C.W.; Xiao, Y.S.; Yuan, Y.F.; Liu, Q.Y.; Liu, Z.S. The long noncoding RNA, EGFR-AS1, a target of GHR, increases the expression of EGFR in hepatocellular carcinoma. *Tumour Biol.* **2015**, *37*, 1079–1089.
15. Liu, J.; Zhang, C.; Lin, M.; Zhu, W.; Liang, Y.; Hong, X.; Zhao, Y.; Young, K.H.; Hu, W.; Feng, Z. Glutaminase 2 negatively regulates the PI3K/AKT signaling and shows tumor suppression activity in human hepatocellular carcinoma. *Oncotarget* **2014**, *5*, 2635–2647.
16. Zhang, J.; Wang, C.; Chen, M.; Cao, J.; Zhong, Y.; Chen, L.; Shen, H.M.; Xia, D. Epigenetic silencing of glutaminase 2 in human liver and colon cancers. *BMC Cancer* **2013**, *13*, 601.
17. Gailhouste, L.; Gomez-Santos, L.; Hagiwara, K.; Hatada, I.; Kitagawa, N.; Kawaharada, K.; Thirion, M.; Kosaka, N.; Takahashi, R.U.; Shibata, T.; *et al.* miR-148a plays a pivotal role in the liver by promoting the hepatospecific phenotype and suppressing the invasiveness of transformed cells. *Hepatology* **2013**, *58*, 1153–1165.
18. Zhou, J.N.; Zeng, Q.; Wang, H.Y.; Zhang, B.; Li, S.T.; Nan, X.; Cao, N.; Fu, C.J.; Yan, X.L.; Jia, Y.L.; *et al.* MicroRNA-125b attenuates epithelial-mesenchymal transitions and targets stem-like liver cancer cells through small mothers against decapentaplegic 2 and 4. *Hepatology* **2015**, *62*, 801–815.
19. Fu, L.; Dong, S.S.; Xie, Y.W.; Tai, L.S.; Chen, L.; Kong, K.L.; Man, K.; Xie, D.; Li, Y.; Cheng, Y.; *et al.* Down-regulation of tyrosine aminotransferase at a frequently deleted region 16q22 contributes to the pathogenesis of hepatocellular carcinoma. *Hepatology* **2010**, *51*, 1624–1634.
20. Jiang, G.; Cui, Y.; Yu, X.; Wu, Z.; Ding, G.; Cao, L. miR-211 suppresses hepatocellular carcinoma by downregulating SATB2. *Oncotarget* **2015**, *6*, 9457–9466.
21. Fan, M.Q.; Huang, C.B.; Gu, Y.; Xiao, Y.; Sheng, J.X.; Zhong, L. Decrease expression of microRNA-20a promotes cancer cell proliferation and predicts poor survival of hepatocellular carcinoma. *J. Exp. Clin. Cancer Res.* **2013**, *32*, 21.
22. Funakoshi, N.; Chaze, I.; Alary, A.S.; Tachon, G.; Cunat, S.; Giansily-Blaizot, M.; Bismuth, M.; Larrey, D.; Pageaux, G.P.; Schved, J.F.; *et al.* The role of genetic factors in patients with hepatocellular carcinoma and iron overload—A prospective series of 234 patients. *Liver Int.* **2015**, *36*, 746–754.
23. Yang, Z.; Zhuang, L.; Szatmary, P.; Wen, L.; Sun, H.; Lu, Y.; Xu, Q.; Chen, X. Upregulation of heat shock proteins (HSPA12A, HSP90B1, HSPA4, HSPA5 and HSPA6) in tumour tissues is associated with poor outcomes from HBV-related early-stage hepatocellular carcinoma. *Int. J. Med. Sci*. **2015**, *12*, 256–263.
24. Wu, J.Q.; Saksena, M.M.; Soriano, V.; Vispo, E.; Saksena, N.K. Differential regulation of cytotoxicity pathway discriminating between HIV, HCV mono- and co-infection identified by transcriptome profiling of PBMCs. *Virol. J.* **2015**, *12*, 4.
25. Xia, H.; Ooi, L.L.; Hui, K.M. MiR-214 targets β-catenin pathway to suppress invasion, stem-like traits and recurrence of human hepatocellular carcinoma. *PLoS ONE* **2012**, *7*, e44206.
26. Tsou, A.P.; Chuang, Y.C.; Su, J.Y.; Yang, C.W.; Liao, Y.L.; Liu, W.K.; Chiu, J.H.; Chou, C.K. Overexpression of a novel imprinted gene, PEG10, in human hepatocellular carcinoma and in regenerating mouse livers. *J. Biomed. Sci.* **2003**, *10 Pt 1*, 625–635.
27. Bang, H.; Ha, S.Y.; Hwang, S.H.; Park, C.K. Expression of PEG10 Is Associated with Poor Survival and Tumor Recurrence in Hepatocellular Carcinoma. *Cancer Res. Treat.* **2015**, *47*, 844–852.
28. Jie, X.; Lang, C.; Jian, Q.; Chaoqun, L.; Dehua, Y.; Yi, S.; Yanping, J.; Luokun, X.; Qiuping, Z.; Hui, W.; *et al.* Androgen activates PEG10 to promote carcinogenesis in hepatic cancer cells. *Oncogene* **2007**, *26*, 5741–5751.
29. Wang, R.; Zhao, N.; Li, S.; Fang, J.H.; Chen, M.X.; Yang, J.; Jia, W.H.; Yuan, Y.; Zhuang, S.M. MicroRNA-195 suppresses angiogenesis and metastasis of hepatocellular carcinoma by inhibiting the ex- pression of VEGF, VAV2, and CDC42. *Hepatology* **2013**, *58*, 642–653.

30. Wang, Y.; Toh, H.C.; Chow, P.; Chung, A.Y.; Meyers, D.J.; Cole, P.A.; Ooi, L.L.; Lee, C.G. MicroRNA-224 is up-regulated in hepatocellular carcinoma through epigenetic mechanisms. *FASEB J.* **2012**, *26*, 3032–3041.
31. Wong, C.M.; Wei, L.; Au, S.L.; Fan, D.N.; Zhou, Y.; Tsang, F.H.; Law, C.T.; Lee, J.M.; He, X.; Shi, J.; *et al*. miR-200b/200c/429 subfamily negatively regulates Rho/ROCK signaling pathway to suppress hepatocellular carcinoma metastasis. *Oncotarget* **2015**, *6*, 13658–13670.
32. Nault, J.C.; Datta, S.; Imbeaud, S.; Franconi, A.; Mallet, M.; Couchy, G.; Letouze, E.; Pilati, C.; Verret, B.; Blanc, J.F.; *et al*. Recurrent AAV2-related insertional mutagenesis in human hepatocellular carcinomas. *Nat. Genet.* **2015**, *47*, 1187–1193.
33. Jain, S.; Singhal, S.; Lee, P.; Xu, R. Molecular genetics of hepatocellular neoplasia. *Am. J. Transl. Res.* **2010**, *2*, 105–118.
34. Wang, B.; Li, D.; Sidler, C.; Rodriguez-Juarez, R.; Singh, N.; Heyns, M.; Ilnytskyy, Y.; Bronson, R.T.; Kovalchuk, O. A suppressive role of ionizing radiation-responsive miR-29c in the development of liver carcinoma via targeting WIP1. *Oncotarget* **2015**, *6*, 9937–9950.
35. Yao, J.; Liang, L.; Huang, S.; Ding, J.; Tan, N.; Zhao, Y.; Yan, M.; Ge, C.; Zhang, Z.; Chen, T.; *et al*. MicroRNA-30d promotes tumor invasion and metastasis by targeting Galphai2 in hepatocellular carcinoma. *Hepatology* **2010**, *51*, 846–856.
36. Wang, W.; Lin, H.; Zhou, L.; Zhu, Q.; Gao, S.; Xie, H.; Liu, Z.; Xu, Z.; Wei, J.; Huang, X.; *et al*. MicroRNA-30a-3p inhibits tumor proliferation, invasiveness and metastasis and is downregulated in hep-atocellular carcinoma. *Eur. J. Surg. Oncol*. **2014**, *40*, 1586–1594.
37. Zhong, K.; Chen, K.; Han, L.; Li, B. MicroRNA-30b/c inhibits non-small cell lung cancer cell prolifer- ation by targeting Rab18. *BMC Cancer* **2014**, *14*, 703.
38. Kim, E.H.; Park, A.K.; Dong, S.M.; Ahn, J.H.; Park, W.Y. Global analysis of CpG methylation reveals epigenetic control of the radiosensitivity in lung cancer cell lines. *Oncogene* **2010**, *29*, 4725–4731.
39. Wang, J.; Qian, J.; Hoeksema, M.D.; Zou, Y.; Espinosa, A.V.; Rahman, S.M.; Zhang, B.; Massion, P.P. Integrative genomics analysis identifies candidate drivers at 3q26-29 amplicon in squamous cell carcinoma of the lung. *Clin. Cancer Res.* **2013**, *19*, 5580–5590.
40. Fujimori, S.; Abe, Y.; Nishi, M.; Hamamoto, A.; Inoue, Y.; Ohnishi, Y.; Nishime, C.; Matsumoto, H.; Mazaki, H.Y.; Kijima, H.; *et al*. The subunits of glutamate cysteine ligase enhance cisplatin resistance in human non-small cell lung cancer xenografts *in vivo*. *Int. J. Oncol.* **2004**, *25*, 413–418.
41. Oshika, Y.; Masuda, K.; Tokunaga, T.; Hatanaka, H.; Kamiya, T.; Abe, Y.; Ozeki, Y.; Kijima, H.; Yamazaki, H.; Tamaoki; *et al*. Thrombospondin 2 gene expression is correlated with decreased vascularity in non-small cell lung cancer. *Clin. Cancer Res.* **1998**, *4*, 1785–1788.
42. Song, N.; Liu, B.; Wu, J.L.; Zhang, R.F.; Duan, L.; He, W.S.; Zhang, C.M. Prognostic value of HMGB3 expression in patients with non-small cell lung cancer. *Tumour Biol.* **2013**, *34*, 2599–2603.
43. Tang, Y.N.; Ding, W.Q.; Guo, X.J.; Yuan, X.W.; Wang, D.M.; Song, J.G. Epigenetic regulation of Smad2 and Smad3 by profilin-2 promotes lung cancer growth and metastasis. *Nat. Commun*. **2015**, *6*, 8230.
44. Nakayama, S.; Soejima, K.; Yasuda, H.; Yoda, S.; Satomi, R.; Ikemura, S.; Terai, H.; Sato, T.; Yamaguchi, N.; Hamamoto, J.; *et al*. FOXD1 expression is associated with poor prognosis in non-small cell lung cancer. *Anticancer Res.* **2015**, *35*, 261–268.
45. Chen, G.; Kim, S.H.; King, A.N.; Zhao, L.; Simpson, R.U.; Christensen, P.J.; Wang, Z.; Thomas, D.G.; Giordano, T.J.; Lin, L.; *et al*. CYP24A1 is an independent prognostic marker of survival in patients with lung adenocarcinoma. *Clin. Cancer Res.* **2011**, *17*, 817–826.
46. Chen, Y.; Knosel, T.; Ye, F.; Pacyna-Gengelbach, M.; Deutschmann, N.; Petersen, I. Decreased PITX1 homeobox gene expression in human lung cancer. *Lung Cancer* **2007**, *55*, 287–294.
47. Chen, J.; Ye, L.; Xie, F.; Yang, Y.; Zhang, L.; Jiang, W.G. Expression of bone morphogenetic protein 7 in lung cancer and its biological impact on lung cancer cells. *Anticancer Res.* **2010**, *30*, 1113–1120.
48. Xiong, D.; Li, G.; Li, K.; Xu, Q.; Pan, Z.; Ding, F.; Vedell, P.; Liu, P.; Cui, P.; Hua, X.; *et al*. Exome sequencing identifies MXRA5 as a novel cancer gene frequently mutated in non-small cell lung carcinoma from Chinese patients. *Carcinogenesis* **2012**, *33*, 1797–1805.
49. Ji, X.; Qian, J.; Rahman, J.; Harris, B.; Hoeksema, M.; Chen, H.; Eisenberg, R.; Young, J. Abstract a10: Slc7a11 contributes to the pathogenesis of lung cancer. *Mol. Cancer Res.* **2016**, *14* (Suppl. 1), A10.
50. Liao, Y.; Ni, Y.; He, R.; Liu, W.; Du, J. Clinical implications of fibroblast activation protein- in non-small cell lung cancer after curative resection: A new predictor for prognosis. *J. Cancer Res. Clin. Oncol.* **2013**, *139*, 1523–1528.

51. Jiang, B.Y.; Zhang, X.C.; Su, J.; Meng, W.; Yang, X.N.; Yang, J.J.; Zhou, Q.; Chen, Z.Y.; Chen, Z.H.; Xie, Z.; *et al*. BCL11A overexpression predicts survival and relapse in non-small cell lung cancer and is modulated by microRNA-30a and gene amplification. *Mol. Cancer* **2013**, *12*, 61.
52. Shao, S.; Li, Z.; Gao, W.; Yu, G.; Liu, D.; Pan, F. ADAM-12 as a diagnostic marker for the proliferation, migration and invasion in patients with small cell lung cancer. *PLoS ONE* **2014**, *9*, e85936.
53. Zakaria, N.; Yusoff, N.M.; Zakaria, Z.; Lim, M.N.; Baharuddin, P.J.; Fakiruddin, K.S.; Yahaya, B. Hu-man non-small cell lung cancer expresses putative cancer stem cell markers and exhibits the transcriptomic profile of multipotent cells. *BMC Cancer* **2015**, *15*, 84.
54. Provencio, M.; Sanchez, A. Therapeutic integration of new molecule-targeted therapies with radio-therapy in lung cancer. *Transl. Lung. Cancer Res*. **2014**, *3*, 89–94.
55. Shen, Z.; Chen, B.; Gan, X.; Hu, W.; Zhong, G.; Li, H.; Xie, X.; Liu, Y.; Li, H.; Xu, X.; *et al*. Methylation of neurofilament light polypeptide promoter is associated with cell invasion and metastasis in NSCLC. *Biochem. Biophys. Res. Commun.* **2016**, *470*, 627–634.
56. Haley, J.A.; Haughney, E.; Ullman, E.; Bean, J.; Haley, J.D.; Fink, M.Y. Altered Transcriptional Control Networks with Trans-Differentiation of Isogenic Mutant-KRas NSCLC Models. *Front. Oncol*. **2014**, *4*, 344.
57. Hou, J.; Lambers, M.; den Hamer, B.; den Bakker, M.A.; Hoogsteden, H.C.; Grosveld, F.; Hegmans, J.; Aerts, J.; Philipsen, S. Expression profiling-based subtyping identifies novel non-small cell lung cancer subgroups and implicates putative resistance to pemetrexed therapy. *J. Thorac. Oncol*. **2012**, *7*, 105–114.
58. Ke, Z.; He, W.; Lai, Y.; Guo, X.; Chen, S.; Li, S.; Wang, Y.; Wang, L. Overexpression of collagen triple helix repeat containing 1 (CTHRC1) is associated with tumour aggressiveness and poor prognosis in human non-small cell lung cancer. *Oncotarget* **2014**, *5*, 9410–9424.
59. Zhang, C.; Zhai, S.; Li, X.; Zhang, Q.; Wu, L.; Liu, Y.; Jiang, C.; Zhou, H.; Li, F.; Zhang, S.; *et al*. Synergistic action by multi-targeting compounds produces a potent compound combination for human NSCLC both in vitro and in vivo. *Cell Death Dis*. **2014**, *5*, e1138.
60. Xia, Y.; Zhu, Y.; Ma, T.; Pan, C.; Wang, J.; He, Z.; Li, Z.; Qi, X.; Chen, Y. miR-204 functions as a tumor suppressor by regulating SIX1 in NSCLC. *FEBS Lett.* **2014**, *588*, 3703–3712.
61. Okabe, N.; Ezaki, J.; Yamaura, T.; Muto, S.; Osugi, J.; Tamura, H.; Imai, J.; Ito, E.; Yanagisawa, Y.; Honma, R.; *et al*. FAM83B is a novel biomarker for diagnosis and prognosis of lung squamous cell carcinoma. *Int. J. Oncol.* **2015**, *46*, 999–1006.
62. Yamashita, R.; Sato, M.; Kakumu, T.; Hase, T.; Yogo, N.; Maruyama, E.; Sekido, Y.; Kondo, M.; Hasegawa, Y. Growth inhibitory effects of miR-221 and miR-222 in non-small cell lung cancer cells. *Cancer Med*. **2015**, *4*, 551–564.
63. Su, Y.; Pan, L. Identification of logic relationships between genes and subtypes of non-small cell lung cancer. *PLoS ONE* **2014**, *9*, e94644.
64. Acunzo, M.; Visone, R.; Romano, G.; Veronese, A.; Lovat, F.; Palmieri, D.; Bottoni, A.; Garofalo, M.; parini, P.G.; Condorelli, G.; *et al*. miR-130a targets MET and induces TRAIL- sensitivity in NSCLC by downregulating miR-221 and 222. *Oncogene* **2012**, *31*, 634–642.
65. Kim, S.J.; Hwang, S.H.; Kim, I.J.; Lee, M.K.; Lee, C.H.; Lee, S.Y.; Lee, E.Y. The association of 18F-deoxyglucose (FDG) uptake of PET with polymorphisms in the glucose transporter gene (SLC2A1) and hypoxia-related genes (HIF1A, VEGFA, APEX1) in non-small cell lung cancer. SLC2A1 polymorphisms and FDG-PET in NSCLC patients. *J. Exp. Clin. Cancer Res.* **2010**, *29*, 69.
66. Watanabe, H.; Imaizumi, M.; Ojika, T.; Abe, T.; Hida, T.; Kato, K. Evaluation of biological character- istics of lung cancer by the human 28 kDa vitamin D-dependent calcium binding protein, calbindin-D28k. *Jpn. J. Clin. Oncol.* **1994**, *24*, 121–127.
67. Iacono, M.L.; Monica, V.; Saviozzi, S.; Ceppi, P.; Bracco, E.; Papotti, M.; Scagliotti, G.V. p63 and p73 isoform expression in non-small cell lung cancer and corresponding morphological normal lung tissue. *J. Thorac. Oncol*. **2011**, *6*, 473–481.
68. Rosen, S.D.; Lemjabbar-Alaoui, H. Sulf-2: An extracellular modulator of cell signaling and a cancer target candidate. *Expert Opin. Ther. Targets* **2010**, *14*, 935–949.
69. Chen, C.H.; Lai, J.M.; Chou, T.Y.; Chen, C.Y.; Su, L.J.; Lee, Y.C.; Cheng, T.S.; Hong, Y.R.; Chou, C.K.; Whang-Peng, J.; *et al*. VEGFA upregulates FLJ10540 and modulates migration and invasion of lung cancer via PI3K/AKT pathway. *PLoS ONE* **2009**, *4*, e5052.

70. Liu, J.; Lu, K.H.; Liu, Z.L.; Sun, M.; De, W.; Wang, Z.X. MicroRNA-100 is a potential molecular marker of non-small cell lung cancer and functions as a tumor suppressor by targeting polo-like kinase 1. *BMC Cancer* **2012**, *12*, 519.
71. Shinmura, K.; Kato, H.; Matsuura, S.; Inoue, Y.; Igarashi, H.; Nagura, K.; Nakamura, S.; Maruyama, K.; Tajima, M.; Funai, K.; *et al*. A novel somatic FGFR3 mutation in primary lung cancer. *Oncol. Rep.* **2014**, *31*, 1219–1224.
72. Nian, W.; Ao, X.; Wu, Y.; Huang, Y.; Shao, J.; Wang, Y.; Chen, Z.; Chen, F.; Wang, D. miR-223 functions as a potent tumor suppressor of the Lewis lung carcinoma cell line by targeting insulin-like growth factor-1 receptor and cyclin-dependent kinase 2. *Oncol. Lett.* **2013**, *6*, 359–366.
73. Justilien, V.; Jameison, L.; Der, C.J.; Rossman, K.L.; Fields, A.P. Oncogenic activity of Ect2 is regulated through protein kinase C iota-mediated phosphorylation. *J. Biol. Chem.* **2011**, *286*, 8149–8157.
74. Xia, H.; Sun, S.; Wang, B.; Wang, T.; Liang, C.; Li, G.; Huang, C.; Qi, D.; Chu, X. miR-143 inhibits NSCLC cell growth and metastasis by targeting Limk1. *Int. J. Mol. Sci.* **2014**, *15*, 11973–11983.
75. Hofmann, H.S.; Bartling, B.; Simm, A.; Murray, R.; Aziz, N.; Hansen, G.; Silber, R.E.; Burdach, S. Identification and classification of differentially expressed genes in non-small cell lung cancer by expression profiling on a global human 59.620-element oligonucleotide array. *Oncol. Rep.* **2006**, *16*, 587–595.
76. Watanabe, T.; Miura, T.; Degawa, Y.; Fujita, Y.; Inoue, M.; Kawaguchi, M.; Furihata, C. Comparison of lung cancer cell lines representing four histopathological subtypes with gene expression profiling using quantitative real-time PCR. *Cancer Cell Int.* **2010**, *10*, 2.
77. Li, X.; Liu, X.; Xu, Y.; Liu, J.; Xie, M.; Ni, W.; Chen, S. KLF5 promotes hypoxia-induced survival and inhibits apoptosis in non-small cell lung cancer cells via HIF-1. *Int. J. Oncol.* **2014**, *45*, 1507–1514.
78. Huang, Y.; Dai, Z.; Barbacioru, C.; Sadee, W. Cystine-glutamate transporter SLC7A11 in cancer chemosensitivity and chemoresistance. *Cancer Res.* **2005**, *65*, 7446–7454.
79. Fu, J.; Khaybullin, R.; Liang, X.; Morin, M.; Xia, A.; Yeh, A.; Qi, X. Discovery of Gene Regulation Pattern in Lung Cancer by Gene Expression Profiling Using Human Tissues. *Genom. Data* **2015**, *3*, 112–115.
80. Chen, R.; Khatri, P.; Mazur, P.K.; Polin, M.; Zheng, Y.; Vaka, D.; Hoang, C.D.; Shrager, J.; Xu, Y.; Vicent, S.; *et al*. A meta-analysis of lung cancer gene expression identifies PTK7 as a survival gene in lung adenocarcinoma. *Cancer Res.* **2014**, *74*, 2892–2902.
81. Chen, Z.; Zeng, H.; Guo, Y.; Liu, P.; Pan, H.; Deng, A.; Hu, J. miRNA-145 inhibits non-small cell lung cancer cell proliferation by targeting c-Myc. *J. Exp. Clin. Cancer Res.* **2010**, *29*, 151.
82. Wangari-Talbot, J.; Hopper-Borge, E. Drug Resistance Mechanisms in Non-Small Cell Lung Carcinoma. *J. Can. Res. Updates* **2013**, *2*, 265–282.
83. Racz, A.; Brass, N.; Heckel, D.; Pahl, S.; Remberger, K.; Meese, E. Expression analysis of genes at 3q26-q27 involved in frequent amplification in squamous cell lung carcinoma. *Eur. J. Cancer* **1999**, *35*, 641–646.
84. Yang, Y.; Wu, J.; Cai, J.; He, Z.; Yuan, J.; Zhu, X.; Li, Y.; Li, M.; Guan, H. PSAT1 regulates cyclin D1 degradation and sustains proliferation of non-small cell lung cancer cells. *Int. J. Cancer* **2015**, *136*, 39–50.
85. Li, Y.; Chao, Y.; Fang, Y.; Wang, J.; Wang, M.; Zhang, H.; Ying, M.; Zhu, X.; Wang, H. MTA1 promotes the invasion and migration of non-small cell lung cancer cells by downregulating miR-125b. *J. Exp. Clin. Cancer Res.* **2013**, *32*, 33.
86. Kren, L.; Goncharuk, V.N.; Krenova, Z.; Stratil, D.; Hermanova, M.; Skrickova, J.; Sheehan, C.E.; Ross, J.S. Expression of matrix metalloproteinases 3, 10 and 11 (stromelysins 1, 2 and 3) and matrix metalloproteinase 7 (matrilysin) by cancer cells in non-small cell lung neoplasms. Clinicopathologic studies. *Cesk Patol.* **2006**, *42*, 16–19.
87. Liu, B.; Qu, J.; Xu, F.; Guo, Y.; Wang, Y.; Yu, H.; Qian, B. MiR-195 suppresses non-small cell lung cancer by targeting CHEK1. *Oncotarget* **2015**, *6*, 9445–9456.
88. Suzuki, C.; Daigo, Y.; Ishikawa, N.; Kato, T.; Hayama, S.; Ito, T.; Tsuchiya, E.; Nakamura, Y. ANLN plays a critical role in human lung carcinogenesis through the activation of RHOA and by involvement in the phosphoinositide 3-kinase/AKT pathway. *Cancer Res.* **2005**, *65*, 11314–11325.
89. Bulk, E.; Ay, A.S.; Hammadi, M.; Ouadid-Ahidouch, H.; Schelhaas, S.; Hascher, A.; Rohde, C.; Thoennissen, N.H.; Wiewrodt, R.; Schmidt, E.; *et al*. Epigenetic dysregulation of KCa 3.1 channels induces poor prognosis in lung cancer. *Int. J. Cancer* **2015**, *137*, 1306–1317.
90. Chang, D.H.; Rutledge, J.R.; Patel, A.A.; Heerdt, B.G.; Augenlicht, L.H.; Korst, R.J. The effect of lung cancer on cytokine expression in peripheral blood mononuclear cells. *PLoS ONE* **2013**, *8*, e64456.

91. Kato, T.; Wada, H.; Patel, P.; Hu, H.P.; Lee, D.; Ujiie, H.; Hirohashi, K.; Nakajima, T.; Sato, M.; Kaji, M.; *et al*. Overexpression of KIF23 predicts clinical outcome in primary lung cancer patients. *Lung Cancer* **2016**, *92*, 53–61.
92. Nishigaki, Y.; Ohsaki, Y.; Toyoshima, E.; Kikuchi, K. Increased serum and urinary levels of a parathy-roid hormone-related protein COOH terminus in non-small cell lung cancer patients. *Clin. Cancer Res.* **1999**, *5*, 1473–1481.
93. Meyer, B.; Loeschke, S.; Schultze, A.; Weigel, T.; Sandkamp, M.; Goldmann, T.; Vollmer, E.; Bullerdiek, J. HMGA2 overexpression in non-small cell lung cancer. *Mol. Carcinog.* **2007**, *46*, 503–511.
94. Gugger, M.; White, R.; Song, S.; Waser, B.; Cescato, R.; Riviere, P.; Reubi, J.C. GPR87 is an overex- pressed G-protein coupled receptor in squamous cell carcinoma of the lung. *Dis. Markers* **2008**, *24*, 41–50.
95. Benlloch, S.; Galbis, J.; Peiro, F.M.; Alenda, C.; Rodriguez-Paniagua, J.M.; Sanchez-Paya, J.; Romero, S.; Ciriquian, J.L.M.; Massuti, B. Role of cea, plunc and ck19 mrna expression in lymph nodes from resected stage i non-small cell lung cancer (nsclc) patients (p) as markers of occult micrometastasis. A pilot study. *J. Clin. Oncol.* **2005**, *23*, 9654.
96. Ji, Y.; Xie, M.; Lan, H.; Zhang, Y.; Long, Y.; Weng, H.; Li, D.; Cai, W.; Zhu, H.; Niu, Y.; *et al*. PRR11 is a novel gene implicated in cell cycle progression and lung cancer. *Int. J. Biochem. Cell Biol.* **2013**, *45*, 645–656.
97. Ziegler, A.; Cerciello, F.; Bigosch, C.; Bausch-Fluck, D.; Felley-Bosco, E.; Ossola, R.; Soltermann, A.; Stahel, R.A.; Wollscheid, B. Proteomic surfaceome analysis of mesothelioma. *Lung Cancer* **2012**, *75*, 189–196.
98. Yang, Y.; Zhang, P.; Zhao, Y.; Yang, J.; Jiang, G.; Fan, J. Decreased MicroRNA-26a expression causes cisplatin resistance in human non-small cell lung cancer. *Cancer Biol. Ther.* **2015**, in press.
99. Zhou, W.; Yin, M.; Cui, H.; Wang, N.; Zhao, L.L.; Yuan, L.Z.; Yang, X.P.; Ding, X.M.; Men, F.Z.; Ma, X.; *et al*. Identification of potential therapeutic target genes and mechanisms in non-small-cell lung carcinoma in non-smoking women based on bioinformatics analysis. *Eur. Rev. Med. Pharmacol. Sci*. **2015**, *19*, 3375–3384.
100. Davidson, A. Plod2 and ttf1 expression as prognostic variables in selection for adjuvant treatment in early stage lung adenocarcinoma. *J. Thorac. Oncol.* **2007**, *2*, S518.
101. Kane, S.; Prentice, M.A.; Mariano, J.M.; Cuttitta, F.; Jakowlew, S.B. Differential induction of early response genes by adrenomedullin and transforming growth factor-beta1 in human lung cancer cells. *Anticancer Res.* **2002**, *22*, 1433–1444.
102. Zhan, P.; Shen, X.K.; Qian, Q.; Zhu, J.P.; Zhang, Y.; Xie, H.Y.; Xu, C.H.; Hao, K.K.; Hu, W.; Xia, N.; *et al*. Down-regulation of lysyl oxidase-like 2 (LOXL2) is associated with disease progression in lung adenocarcinomas. *Med. Oncol.* **2012**, *29*, 648–655.
103. Wang, K.K.; Liu, N.; Radulovich, N.; Wigle, D.A.; Johnston, M.R.; Shepherd, F.A.; Minden, M.D.; Tsao, M.S. Novel candidate tumor marker genes for lung adenocarcinoma. *Oncogene* **2002**, *21*, 7598–7604.
104. Sanchez-Palencia, A.; Gomez-Morales, M.; Gomez-Capilla, J.A.; Pedraza, V.; Boyero, L.; Rosell, R.; Farez-Vidal, M.E. Gene expression profiling reveals novel biomarkers in nonsmall cell lung cancer. *Int. J. Cancer* **2011**, *129*, 355–364.
105. Yao, L.; Sun, B.; Zhao, X.; Zhao, X.; Gu, Q.; Dong, X.; Zheng, Y.; Sun, J.; Cheng, R.; Qi, H.; *et al*. Overexpression of Wnt5a promotes angiogenesis in NSCLC. *BioMed Res. Int*. **2014**, *2014*, 832562.
106. Jin, M.M.; Ye, Y.Z.; Qian, Z.D.; Zhang, Y.B. Notch signaling molecules as prognostic biomarkers for non-small cell lung cancer. *Oncol. Lett*. **2015**, *10*, 3252–3260.
107. Chow, G.; Tauler, J.; Mulshine, J.L. Cytokines and growth factors stimulate hyaluronan production: Role of hyaluronan in epithelial to mesenchymal-like transition in non-small cell lung cancer. *J. Biomed. Biotechnol.* **2010**, *2010*, 485468.
108. Fabbri, M.; Garzon, R.; Cimmino, A.; Liu, Z.; Zanesi, N.; Callegari, E.; Liu, S.; Alder, H.; Costinean, S.; Fernandez-Cymering, C.; *et al*. MicroRNA-29 family reverts aberrant methylation in lung cancer by targeting DNA methyltransferases 3A and 3B. *Proc. Natl. Acad. Sci. USA* **2007**, *104*, 15805–15810.
109. Lonergan, K.M.; Chari, R.; Coe, B.P.; Wilson, I.M.; Tsao, M.S.; Ng, R.T.; Macaulay, C.; Lam, S.; Lam, W.L. Transcriptome profiles of carcinoma-in-situ and invasive non-small cell lung cancer as revealed by SAGE. *PLoS ONE* **2010**, *5*, e9162.
110. Savci-Heijink, C.D.; Kosari, F.; Aubry, M.C.; Caron, B.L.; Sun, Z.; Yang, P.; Vasmatzis, G. The role of desmoglein-3 in the diagnosis of squamous cell carcinoma of the lung. *Am. J. Pathol.* **2009**, *174*, 1629–1637.
111. Guan, P.; Huang, D.; He, M.; Zhou, B. Lung cancer gene expression database analysis incorporating prior knowledge with support vector machine-based classification method. *J. Exp. Clin. Cancer Res.* **2009**, *28*, 103.

112. Xie, X.; Liu, H.T.; Mei, J.; Ding, F.B.; Xiao, H.B.; Hu, F.Q.; Hu, R.; Wang, M.S. miR-106a promotes growth and metastasis of non-small cell lung cancer by targeting PTEN. *Int. J. Clin. Exp. Pathol.* **2015**, *8*, 3827–3834.
113. Osada, H.; Takahashi, T. let-7 and miR-17-92: small-sized major players in lung cancer development. *Cancer Sci.* **2011**, *102*, 9–17.
114. Lin, J.; Marquardt, G.; Mullapudi, N.; Wang, T.; Han, W.; Shi, M.; Keller, S.; Zhu, C.; Locker, J.; Spivack, S.D. Lung cancer transcriptomes refined with laser capture microdissection. *Am. J. Pathol.* **2014**, *184*, 2868–2884.
115. Song, Y.F.; Hong, J.F.; Liu, D.L.; Lin, Q.A.; Lan, X.P.; Lai, G.X. miR-630 targets LMO3 to regulate cell growth and metastasis in lung cancer. *Am. J. Transl. Res.* **2015**, *7*, 1271–1279.
116. Cao, C.; Zhang, M.; Gao, R.; Cress, D.; Chen, Z.; Wu, L.; Kaye, M.Z.; Kaye, F. Abstract 3105: Orphan nuclear receptor nr4a2 exhibits oncogenic activity in lung cancer cells. *Cancer Res.* **2013**, *73* (Suppl. 8), 3105.
117. Xinjian Wang, David Balli, Tien Le, Vladimir V. Kalinichenko, and Tatiana V. Kalin. Abstract a60: Foxf1 transcription factor drives progression of benign lung adenomas to malignant adenocarcinomas. *Cancer Res.* **2013**, *73* (Suppl. 3), A60.
118. Jeon, H.S.; Dracheva, T.; Yang, S.H.; Meerzaman, D.; Fukuoka, J.; Shakoori, A.; Shilo, K.; Travis, W.D.; , J. SMAD6 contributes to patient survival in non-small cell lung cancer and its knockdown reestablishes TGF-beta homeostasis in lung cancer cells. *Cancer Res.* **2008**, *68*, 9686–9692.
119. Carrega, P.; Morandi, B.; Costa, R.; Frumento, G.; Forte, G.; Altavilla, G.; Ratto, G.B.; Mingari, M.C.; Moretta, L.; Ferlazzo, G. Natural killer cells infiltrating human nonsmall-cell lung cancer are enriched in CD56 bright CD16(-) cells and display an impaired capability to kill tumor cells. *Cancer* **2008**, *112*, 863–875.
120. Healy, K.D.; Hodgson, L.; Kim, T.Y.; Shutes, A.; Maddileti, S.; Juliano, R.L.; Hahn, K.M.; Harden, T.K.; Bang, Y.J.; der, C.J. DLC-1 suppresses non-small cell lung cancer growth and invasion by RhoGAP-dependent and independent mechanisms. *Mol. Carcinog.* **2008**, *47*, 326–337.
121. Liu, N.; Dong, Q.-Z.; Wang, E.-H. Expression of tbx3 is correlated with tumor proliferation and invasion in non-small cell lung cancer. *J. China Med. Univ.* **2012**, *41*, 360.
122. Jiang, C.P.; Wu, B.H.; Chen, S.P.; Fu, M.Y.; Yang, M.; Liu, F.; Wang, B.Q. High COL4A3 expression correlates with poor prognosis after cisplatin plus gemcitabine chemotherapy in non-small cell lung cancer. *Tumour Biol.* **2013**, *34*, 415–420.
123. Kim. G.J. Characterization and Regulation of Stearoyl-CoA Desaturase 5. Ph.D. Thesis, The State University of New Jersey, New Brunswick, NJ, USA, October 2011.
124. Liu, J.; Lee, W.; Jiang, Z.; Chen, Z.; Jhunjhunwala, S.; Haverty, P.M.; Gnad, F.; Guan, Y.; Gilbert, H.N.; Stinson, J.; *et al*. Genome and transcriptome sequencing of lung cancers reveal diverse mutational and splicing events. *Genome Res.* **2012**, *22*, 2315–2327.
125. Sakashita, H.; Inoue, H.; Akamine, S.; Ishida, T.; Inase, N.; Shirao, K.; Mori, M.; Mimori, K. Identification of the NEDD4L gene as a prognostic marker by integrated microarray analysis of copy number and gene expression profiling in non-small cell lung cancer. *Ann. Surg. Oncol.* **2013**, *20* (Suppl. 3), S590–S598.
126. Rudin, C.M.; Durinck, S.; Stawiski, E.W.; Poirier, J.T.; Modrusan, Z.; Shames, D.S.; Bergbower, E.A.; Guan, Y.; Shin, J.; Guillory, J.; *et al*. Comprehensive genomic analysis identifies SOX2 as a frequently amplified gene in small-cell lung cancer. *Nat. Genet.* **2012**, *44*, 1111–1116.
127. Tejero, R.; Navarro, A.; Campayo, M.; Vinolas, N.; Marrades, R.M.; Cordeiro, A.; Ruiz-Martinez, M.; tasusagna, S.S.; Molins, L.; Ramirez, J.; *et al.* miR-141 and miR-200c as markers of overall survival in early stage non-small cell lung cancer adenocarcinoma. *PLoS ONE* **2014**, *9*, e101899.
128. Pacurari, M.; Addison, J.B.; Bondalapati, N.; Wan, Y.W.; Luo, D.; Qian, Y.; Castranova, V.; Ivanov, A.V.; Guo, N.L. The microRNA-200 family targets multiple non-small cell lung cancer prognostic markers in H1299 cells and BEAS-2B cells. *Int. J. Oncol.* **2013**, *43*, 548–560.
129. Tan, X.; Chen, M. MYLK and MYL9 expression in non-small cell lung cancer identified by bioinfor-matics analysis of public expression data. *Tumour Biol.* **2014**, *35*, 12189–12200.
130. Li, Y.; Zhang, D.; Chen, C.; Ruan, Z.; Li, Y.; Huang, Y. MicroRNA-212 displays tumor-promoting properties in non-small cell lung cancer cells and targets the hedgehog pathway receptor PTCH1. *Mol. Biol. Cell* **2012**, *23*, 1423–1434.
131. Cheung, W.K.; Zhao, M.; Liu, Z.; Stevens, L.E.; Cao, P.D.; Fang, J.E.; Westbrook, T.F.; Nguyen, D.X. Control of alveolar differentiation by the lineage transcription factors GATA6 and HOPX inhibits lung adenocarcinoma metastasis. *Cancer Cell* **2013**, *23*, 725–738.

132. Basseres, D.S.; D'Alo, F.; Yeap, B.Y.; Lowenberg, E.C.; Gonzalez, D.A.; Yasuda, H.; Dayaram, T.; Kocher, O.N.; Godleski, J.J.; Richards, W.G.; *et al*. Frequent downregulation of the transcription factor Foxa2 in lung cancer through epigenetic silencing. *Lung Cancer* **2012**, *77*, 31–37.
133. Dong, W.; Yao, C.; Teng, X.; Chai, J.; Yang, X.; Li, B. MiR-140-3p suppressed cell growth and invasion by downregulating the expression of ATP8A1 in non-small cell lung cancer. *Tumour Biol.* **2015**, *37*, 2973–2985.
134. Urgard, E.; Vooder, T.; Vosa, U.; Valk, K.; Liu, M.; Luo, C.; Hoti, F.; Roosipuu, R.; Annilo, T.; Laine, J.; *et al*. Metagenes associated with survival in non-small cell lung cancer. *Cancer Inform*. **2011**, *10*, 175–183.
135. Liu, H.-X.; Bai, Y.-F.; Wu, X.-X.; Guo, L.-L. Expressions and clinical significance of protocadherin 9 and cyclin d1 in non-small cell lung cancer patients. *Chin. J. Cancer Prev. Treat.* **2014**, *21*, doi:10.1515/cclm-2015-0776.
136. Balgkouranidou, I.; Chimonidou, M.; Milaki, G.; Tsaroucha, E.; Kakolyris, S.; Georgoulias, V.; Lianidou, E. SOX17 promoter methylation in plasma circulating tumor DNA of patients with non-small cell lung cancer. *Clin. Chem. Lab. Med.* **2016**, doi:10.1515/cclm-2015-0776.
137. Nasarre, P.; Potiron, V.; Drabkin, H.; Roche, J. Guidance molecules in lung cancer. *Cell Adh Migr*. **2010**, *4*, 130–145.
138. Jones, D.R.; Moskaluk, C.A.; Gillenwater, H.H.; Petroni, G.R.; Burks, S.G.; Philips, J.; Rehm, P.K.; Olazagasti, J.; Kozower, B.D.; Bao, Y. Phase I trial of induction histone deacetylase and proteasome inhibition followed by surgery in non-small-cell lung cancer. *J. Thorac. Oncol*. **2012**, *7*, 1683–1690.
139. Li, M.; Qiu, M.; Xu, Y.; Mao, Q.; Wang, J.; Dong, G.; Xia, W.; Yin, R.; Xu, L. Differentially expressed protein-coding genes and long noncoding RNA in early-stage lung cancer. *Tumour Biol.* **2015**, *36*, 9969–9978.
140. Wang, H.M.; Liao, Z.X.; Komaki, R.; Welsh, J.W.; O'Reilly, M.S.; Chang, J.Y.; Zhuang, Y.; Levy, L.B.; Lu, C.; Gomez, D.R. Improved survival outcomes with the incidental use of beta-blockers among patients with non-small-cell lung cancer treated with definitive radiation therapy. *Ann. Oncol.* **2013**, *24*, 1312–1319.
141. Li, J.; Tan, Q.; Yan, M.; Liu, L.; Lin, H.; Zhao, F.; Bao, G.; Kong, H.; Ge, C.; Zhang, F.; *et al*. miRNA-200c inhibits invasion and metastasis of human non-small cell lung cancer by directly targeting ubiquitin specific peptidase 25. *Mol. Cancer* **2014**, *13*, 166.
142. Moncho-Amor, V.; de Caceres, I.I.; Bandres, E.; Martinez-Poveda, B.; Orgaz, J.L.; Sanchez-Perez, I.; Zazo, S.; Rovira, A.; Albanell, J.; Jimenez, B.; *et al*. DUSP1/MKP1 promotes angiogenesis, invasion and metastasis in non-small-cell lung cancer. *Oncogene* **2011**, *30*, 668–678.
143. Niu, C.; Liang, C.; Guo, J.; Cheng, L.; Zhang, H.; Qin, X.; Zhang, Q.; Ding, L.; Yuan, B.; Xu, X.; *et al*. Downregulation and growth inhibitory role of FHL1 in lung cancer. *Int. J. Cancer* **2012**, *130*, 2549–2556.
144. Song, S.; Walter, V.; Karaca, M.; Li, Y.; Bartlett, C.S.; Smiraglia, D.J.; Serber, D.; Sproul, C.D.; Plass, C.; Zhang, J.; *et al*. Gene silencing associated with SWI/SNF complex loss during NSCLC development. *Mol. Cancer Res.* **2014**, *12*, 560–570.
145. Han, N.; Yuan, X.; Wu, H.; Xu, H.; Chu, Q.; Guo, M.; Yu, S.; Chen, Y.; Wu, K. DACH1 inhibits lung adenocarcinoma invasion and tumor growth by repressing CXCL5 signaling. *Oncotarget* **2015**, *6*, 5877–5888.
146. Hasan, A.N.; Ahmad, M.W.; Madar, I.H.; Grace, B.L.; Hasan, T.N. An in silico analytical study of lung cancer and smokers datasets from gene expression omnibus (GEO) for prediction of differentially expressed genes. *Bioinformation* **2015**, *11*, 229–235.
147. Chen, L.; Zhuo, D.; Chen, J.; Yuan, H. Screening feature genes of lung carcinoma with DNA microarray analysis. *Int. J. Clin. Exp. Med*. **2015**, *8*, 12161–12171.
148. Vaira, V.; Faversani, A.; Martin, N.M.; Garlick, D.S.; Ferrero, S.; Nosotti, M.; Kissil, J.L.; Bosari, S.; Altieri, D.C. Regulation of lung cancer metastasis by Klf4-Numb-like signaling. *Cancer Res.* **2013**, *73*, 2695–2705.
149. Pandiri, A.R.; Sills, R.C.; Ziglioli, V.; Ton, T.V.; Hong, H.H.; Lahousse, S.A.; Gerrish, K.E.; Auerbach, S.S.; Shockley, K.R.; Bushel, P.R.; *et al*. Differential transcriptomic analysis of spontaneous lung tumors in B6C3F1 mice: comparison to human non-small cell lung cancer. *Toxicol. Pathol*. **2012**, *40*, 1141–1159.
150. Selamat, S.A.; Chung, B.S.; Girard, L.; Zhang, W.; Zhang, Y.; Campan, M.; Siegmund, K.D.; Koss, M.N.; Hagen, J.A.; Lam, W.L.; *et al*. Genome-scale analysis of DNA methylation in lung adenocarcinoma and integration with mRNA expression. *Genome Res.* **2012**, *22*, 1197–1211.
151. Cai, J.; Fang, L.; Huang, Y.; Li, R.; Yuan, J.; Yang, Y.; Zhu, X.; Chen, B.; Wu, J.; Li, M. miR-205 targets PTEN and PHLPP2 to augment AKT signaling and drive malignant phenotypes in non-small cell lung cancer. *Cancer Res.* **2013**, *73*, 5402–5415.
152. Wu, M.; Tu, T.; Huang, Y.; Cao, Y. Suppression subtractive hybridization identified differentially expressed genes in lung adenocarcinoma: ERGIC3 as a novel lung cancer-related gene. *BMC Cancer* **2013**, 13, 44.

153. Yang, Y.; Meng, H.; Peng, Q.; Yang, X.; Gan, R.; Zhao, L.; Chen, Z.; Lu, J.; Meng, Q.H. Downregulation of microRNA-21 expression restrains non-small cell lung cancer cell proliferation and migration through upregulation of programmed cell death 4. *Cancer Gene Ther.* **2015**, *22*, 23–29.
154. Tomida, S.; Koshikawa, K.; Yatabe, Y.; Harano, T.; Ogura, N.; Mitsudomi, T.; Some, M.; Yanagisawa, K.; Takahashi, T.; Osada, H.; *et al*. Gene expression-based, individualized outcome prediction for surgically treated lung cancer patients. *Oncogene* **2004**, *23*, 5360–5370.
155. Jiang, L.; Jiang, S.; Lin, Y.; Yang, H.; Zhao, Z.; Xie, Z.; Lin, Y.; Long, H. Combination of body mass index and oxidized low density lipoprotein receptor 1 in prognosis prediction of patients with squamous non-small cell lung cancer. *Oncotarget* **2015**, *6*, 22072–22080.
156. Hayano, T.; Garg, M.; Yin, D.; Sudo, M.; Kawamata, N.; Shi, S.; Chien, W.; Ding, L.W.; Leong, G.; Mori, S.; *et al*. SOX7 is down-regulated in lung cancer. *J. Exp. Clin. Cancer Res.* **2013**, *32*, 17.
157. Wu, C.; Cao, Y.; He, Z.; He, J.; Hu, C.; Duan, H.; Jiang, J. Serum levels of miR-19b and miR-146a as prognostic biomarkers for non-small cell lung cancer. *Tohoku J. Exp. Med.* **2014**, *232*, 85–95.
158. Park, K.S.; Kim, H.K.; Lee, J.H.; Choi, Y.B.; Park, S.Y.; Yang, S.H.; Kim, S.Y.; Hong, K.M. Transglutaminase 2 as a cisplatin resistance marker in non-small cell lung cancer. *J. Cancer Res. Clin. Oncol.* **2010**, *136*, 493–502.
159. Chen, S.C.; Lin, C.Y.; Chen, Y.H.; Fang, H.Y.; Cheng, C.Y.; Chang, C.W.; Chen, R.A.; Tai, H.L.; Lee, C.H.; Chou, M.C.; *et al*. Aberrant promoter methylation of EDNRB in lung cancer in Taiwan. *Oncol. Rep.* **2006**, *15*, 167–172.
160. Karlsson, T.; Kvarnbrink, S.; Holmlund, C.; Botling, J.; Micke, P.; Johansson, M.; Henriksson, R.; Hedman, H. Abstract 5315: Interactions between lrig proteins and lmo7 and the expression of lmo7 in human lung cancer. *Cancer Res.* **2013**, *73* (Suppl. 8), 5315.
161. Zhan, Q.; Huang, R.F.; Liang, X.H.; Ge, M.X.; Jiang, J.W.; Lin, H.; Zhou, X.L. FRAS1 knockdown reduces A549 cells migration and invasion through downregulation of FAK signaling. *Int. J. Clin. Exp. Med*. **2014**, *7*, 1692–1697.
162. Zhang, K.; Dai, L.; Zhang, B.; Xu, X.; Shi, J.; Fu, L.; Chen, X.; Li, J.; Bai, Y. miR-203 is a direct transcrip- tional target of E2F1 and causes G1 arrest in esophageal cancer cells. *J. Cell. Physiol.* **2015**, *230*, 903–910.
163. Guo, Y.; Chen, Z.; Zhang, L.; Zhou, F.; Shi, S.; Feng, X.; Li, B.; Meng, X.; Ma, X.; Luo, M.; *et al*. Distinctive microRNA profiles relating to patient survival in esophageal squamous cell carcinoma. *Cancer Res.* **2008**, *68*, 26–33.
164. Li, C.; Li, Z.; Zhu, M.; Zhao, T.; Chen, L.; Ji, W.; Chen, H.; Su, C. Clinicopathological and prognostic significance of survivin over-expression in patients with esophageal squamous cell carcinoma: A meta-analysis. *PLoS ONE* **2012**, *7*, e44764.
165. Thepot, A.; Hautefeuille, A.; Cros, M.P.; Abedi-Ardekani, B.; Petre, A.; Damour, O.; Krutovskikh, V.; Hainaut, P. Intraepithelial p63-dependent expression of distinct components of cell adhesion complexes in normal esophageal mucosa and squamous cell carcinoma. *Int. J. Cancer* **2010**, *127*, 2051–2062.
166. Wu, C.; Li, M.; Hu, C.; Duan, H. Clinical significance of serum miR-223, miR-25 and miR-375 in patients with esophageal squamous cell carcinoma. *Mol. Biol. Rep.* **2014**, *41*, 1257–1266.
167. Kurashige, J.; Watanabe, M.; Iwatsuki, M.; Kinoshita, K.; Saito, S.; Hiyoshi, Y.; Kamohara, H.; Baba, Y.; Mimori, K.; Baba, H. Overexpression of microRNA-223 regulates the ubiquitin ligase FBXW7 in oesophageal squamous cell carcinoma. *Br. J. Cancer* **2012**, *106*, 182–188.
168. Sawada, G.; Niida, A.; Hirata, H.; Komatsu, H.; Uchi, R.; Shimamura, T.; Takahashi, Y.; Kurashige, J.; Matsumura, T.; Ueo, H.; *et al*. An Integrative Analysis to Identify Driver Genes in Esophageal Squamous Cell Carcinoma. *PLoS ONE* **2015**, *10*, e0139808.
169. Sun, M.; Ju, H.; Zhou, Z.; Zhu, R. Pilot genome-wide study of tandem 3′ UTRs in esophageal cancer using high-throughput sequencing. *Mol. Med. Rep*. **2014**, *9*, 1597–1605.
170. Rane, J.K.; Scaravilli, M.; Ylipaa, A.; Pellacani, D.; Mann, V.M.; Simms, M.S.; Nykter, M.; Collins, A.T.; Visakorpi; Maitland, N.J. MicroRNA expression profile of primary prostate cancer stem cells as a source of biomarkers and therapeutic targets. *Eur. Urol.* **2015**, *67*, 7–10.
171. Cencic, R.; Carrier, M.; Galicia-Vazquez, G.; Bordeleau, M.E.; Sukarieh, R.; Bourdeau, A.; Brem, B.; Teodoro, J.G.; Greger, H.; Tremblay, M.L.; *et al*. Antitumor activity and mechanism of action of the cyclopenta[b]benzofuran, silvestrol. *PLoS ONE* **2009**, *4*, e5223.
172. Miller, G.J.; Miller, H.L.; van Bokhoven, A.; Lambert, J.R.; Werahera, P.N.; Schirripa, O.; Lucia, M.S.; Nordeen, S.K. Aberrant HOXC expression accompanies the malignant phenotype in human prostate. *Cancer Res.* **2003**, *63*, 5879–5888.

173. Larne, O.; Hagman, Z.; Lilja, H.; Bjartell, A.; Edsjo, A.; Ceder, Y. miR-145 suppress the androgen receptor in prostate cancer cells and correlates to prostate cancer prognosis. *Carcinogenesis* **2015**, *36*, 858–866.
174. Johnson, I.R.; Parkinson-Lawrence, E.J.; Shandala, T.; Weigert, R.; Butler, L.M.; Brooks, D.A. Altered endosome biogenesis in prostate cancer has biomarker potential. *Mol. Cancer Res.* **2014**, *12*, 1851–1862.
175. Tsuchiya, N.; Izumiya, M.; Ogata-Kawata, H.; Okamoto, K.; Fujiwara, Y.; Nakai, M.; Okabe, A.; Schetter, A.J.; Bowman, E.D.; Midorikawa, Y.; *et al*. Tumor suppressor miR-22 determines p53-dependent cellular fate through post-transcriptional regulation of p21. *Cancer Res.* **2011**, *71*, 4628–4639.
176. Phin, S.; Moore, M.W.; Cotter, P.D. Genomic Rearrangements of PTEN in Prostate Cancer. *Front. Oncol.* **2013**, *3*, 240.
177. Shen, P.F.; Chen, X.Q.; Liao, Y.C.; Chen, N.; Zhou, Q.; Wei, Q.; Li, X.; Wang, J.; Zeng, H. MicroRNA-494-3p targets CXCR4 to suppress the proliferation, invasion, and migration of prostate cancer. *Prostate* **2014**, *74*, 756–767.
178. Wagner, S.; Ngezahayo, A.; Escobar, H.M.; Nolte, I. Role of miRNA let-7 and its major targets in prostate cancer. *BioMed Res. Int*. **2014**, *2014*, 376326.
179. Takayama, K.; Suzuki, T.; Tsutsumi, S.; Fujimura, T.; Takahashi, S.; Homma, Y.; Urano, T.; Aburatani, H.; Inoue, S. Integrative analysis of FOXP1 function reveals a tumor-suppressive effect in prostate cancer. *Mol. Endocrinol.* **2014**, *28*, 2012–2024.
180. Pang, Y.; Young, C.Y.; Yuan, H. MicroRNAs and prostate cancer. *Acta Biochim. Biophys. Sin. (Shanghai)* **2010**, *42*, 363–369.
181. Fukuhara, H.; Kuramochi, M.; Fukami, T.; Kasahara, K.; Furuhata, M.; Nobukuni, T.; Maruyama, T.; Gai, K.I.; Sekiya, T.; Shuin, T.; *et al*. Promoter methylation of TSLC1 and tumor suppression by its gene product in human prostate cancer. *Jpn. J. Cancer Res.* **2002**, *93*, 605–609.
182. Pesta, M.; Klecka, J.; Kulda, V.; Topolcan, O.; Hora, M.; Eret, V.; Ludvikova, M.; Babjuk, M.; Novak, K.; Stolz, J.; *et al*. Importance of miR-20a expression in prostate cancer tissue. *Anticancer Res.* **2010**, *30*, 3579–3583.
183. Cekaite, L.; Rantala, J.K.; Bruun, J.; Guriby, M.; Agesen, T.H.; Danielsen, S.A.; Lind, G.E.; Nesbakken, A.; Kallioniemi; Lothe, R.A.; *et al.* MiR-9, -31, and -182 deregulation promote proliferation and tumor cell survival in colon cancer. *Neoplasia* **2012**, *14*, 868–879.
184. Meeh, P.F.; Farrell, C.L.; Croshaw, R.; Crimm, H.; Miller, S.K.; Oroian, D.; Kowli, S.; Zhu, J.; Carver, W.; Wu, W.; *et al*. A gene expression classifier of node-positive colorectal cancer. *Neoplasia* **2009**, *11*, 1074–1083.
185. Zhou, T.; Zhang, G.J.; Zhou, H.; Xiao, H.X.; Li, Y. Overexpression of microRNA-183 in human colorectal cancer and its clinical significance. *Eur. J. Gastroenterol. Hepatol*. **2014**, *26*, 229–233.
186. Nakamura, J.; Aoyagi, S.; Nanchi, I.; Nakatsuka, S.; Hirata, E.; Shibata, S.; Fukuda, M.; Yamamoto, Y.; Fukuda, I.; Tatsumi, N.; *et al*. Overexpression of eukaryotic elongation factor eEF2 in gastrointestinal cancers and its involvement in G2/M progression in the cell cycle. *Int. J. Oncol.* **2009**, *34*, 1181–1189.
187. Xu, X.M.; Qian, J.C.; Deng, Z.L.; Cai, Z.; Tang, T.; Wang, P.; Zhang, K.H.; Cai, J.P. Expression of miR-21, miR-31, miR-96 and miR-135b is correlated with the clinical parameters of colorectal cancer. *Oncol. Lett*. **2012**, *4*, 339–345.
188. Dong, Y.; Zhao, J.; Wu, C.W.; Zhang, L.; Liu, X.; Kang, W.; Leung, W.W.; Zhang, N.; Chan, F.K.; Sung, J.J.; *et al*. Tumor suppressor functions of miR-133a in colorectal cancer. *Mol. Cancer Res.* **2013**, *11*, 1051–1060.
189. Asbagh, L.A.; Vazquez, I.; Vecchione, L.; Budinska, E.; de Vriendt, V.; Baietti, M.F.; Steklov, M.; Jacobs, B.; Hoe, N.; Singh, S.; *et al*. The tyrosine phosphatase PTPRO sensitizes colon cancer cells to anti-EGFR therapy through activation of SRC-mediated EGFR signaling. *Oncotarget* **2014**, *5*, 10070–10083.
190. Smith, A.R.; Marquez, R.T.; Tsao, W.C.; Pathak, S.; Roy, A.; Ping, J.; Wilkerson, B.; Lan, L.; Meng, W.; Neufeld, L.; *et al*. Tumor suppressive microRNA-137 negatively regulates Musashi-1 and colorectal cancer progression. *Oncotarget* **2015**, *6*, 12558–12573.
191. Wang, F.; Ma, Y.L.; Zhang, P.; Shen, T.Y.; Shi, C.Z.; Yang, Y.Z.; Moyer, M.P.; Zhang, H.Z.; Chen, H.Q.; Liang, Y.; *et al*. SP1 mediates the link between methylation of the tumour suppressor miR-149 and outcome in colorectal cancer. *J. Pathol.* **2013**, *229*, 12–24.
192. Fan, J.; Yan, D.; Teng, M.; Tang, H.; Zhou, C.; Wang, X.; Li, D.; Qiu, G.; Peng, Z. Digital transcript profile analysis with aRNA-LongSAGE validates FERMT1 as a potential novel prognostic marker for colon cancer. *Clin. Cancer Res.* **2011**, *17*, 2908–2918.
193. Zhang, Q.; Tang, Q.; Qin, D.; Yu, L.; Huang, R.; Lv, G.; Zou, Z.; Jiang, X.C.; Zou, C.; Liu, W.; *et al*. Role of microRNA 30a targeting insulin receptor substrate 2 in colorectal tumorigenesis. *Mol. Cell. Biol.* **2015**, *35*, 988–1000.

194. Wang, R.J.; Wu, P.; Cai, G.X.; Wang, Z.M.; Xu, Y.; Peng, J.J.; Sheng, W.Q.; Lu, H.F.; Cai, S.J. Down-regulated MYH11 expression correlates with poor prognosis in stage II and III colorectal cancer. *Asian Pac. J. Cancer Prev.* **2014**, *15*, 7223–7228.
195. Arabzadeh, A.; Beauchemin, N. Stromal CEACAM1 expression regulates colorectal cancer metastasis. *Oncoimmunology* **2012**, *1*, 1205–1207.
196. Yu, F.; Yao, H.; Zhu, P.; Zhang, X.; Pan, Q.; Gong, C.; Huang, Y.; Hu, X.; Su, F.; Lieberman, J.; *et al*. let-7 regulates self renewal and tumorigenicity of breast cancer cells. *Cell* **2007**, *131*, 1109–1123.
197. Yu, K.; Lee, C.H.; Tan, P.H.; Tan, P. Conservation of breast cancer molecular subtypes and transcrip- tional patterns of tumor progression across distinct ethnic populations. *Clin. Cancer Res.* **2004**, *10*, 5508–5517.
198. Gebre-Medhin, M.; Kindblom, L.G.; Wennbo, H.; Tornell, J.; Meis-Kindblom, J.M. Growth hormone receptor is expressed in human breast cancer. *Am. J. Pathol.* **2001**, *158*, 1217–1222.
199. Gilbert, P.M.; Mouw, J.K.; Unger, M.A.; Lakins, J.N.; Gbegnon, M.K.; Clemmer, V.B.; Benezra, M.; Licht, D.; Boudreau, N.J.; Tsai, K.K.; *et al*. HOXA9 regulates BRCA1 expression to modulate human breast tumor phenotype. *J. Clin. Investig.* **2010**, *120*, 1535–1550.
200. Xue, J.; Chen, Z.; Gu, X.; Zhang, Y.; Zhang, W. MicroRNA-148a inhibits migration of breast cancer cells by targeting MMP-13. *Tumour Biol.* **2015**, *37*, 1581–1590.
201. Alkaied, H.; Harris, K.; Brenner, A.; Awasum, M.; Varma, S. Does hormonal therapy have a therapeutic role in metastatic primary small cell neuroendocrine breast carcinoma? Case report and literature review. *Clin. Breast Cancer* **2012**, *12*, 226–230.
202. Milde-Langosch, K.; Kappes, H.; Riethdorf, S.; Loning, T.; Bamberger, A.M. FosB is highly expressed in normal mammary epithelia, but down-regulated in poorly differentiated breast carcinomas. *Breast Cancer Res. Treat.* **2003**, *77*, 265–275.
203. Chiquet-Ehrismann, R.; Tucker, R.P. Tenascins and the importance of adhesion modulation. *Cold Spring Harb. Perspect. Biol.* **2011**, *3*, doi:10.1101/cshperspect.a004960.
204. Zhang, X.; Yu, H.; Lou, J.R.; Zheng, J.; Zhu, H.; Popescu, N.I.; Lupu, F.; Lind, S.E.; Ding, W.Q. MicroRNA-19 (miR-19) regulates tissue factor expression in breast cancer cells. *J. Biol. Chem.* **2011**, *286*, 1429–1435.
205. Kadota, M.; Sato, M.; Duncan, B.; Ooshima, A.; Yang, H.H.; Diaz-Meyer, N.; Gere, S.; Kageyama, S.; Fukuoka, J.; Nagata, T.; *et al*. Identification of novel gene amplifications in breast cancer and coexistence of gene amplification with an activating mutation of PIK3CA. *Cancer Res.* **2009**, *69*, 7357–7365.
206. Sun, Y.; Mao, X.; Fan, C.; Liu, C.; Guo, A.; Guan, S.; Jin, Q.; Li, B.; Yao, F.; Jin, F. CXCL12-CXCR4 axis promotes the natural selection of breast cancer cell metastasis. *Tumour Biol.* **2014**, *35*, 7765–7773.
207. Planche, A.; Bacac, M.; Provero, P.; Fusco, C.; Delorenzi, M.; Stehle, J.C.; Stamenkovic, I. Identification of prognostic molecular features in the reactive stroma of human breast and prostate cancer. *PLoS ONE* **2011**, *6*, e18640.
208. Yan, L.X.; Huang, X.F.; Shao, Q.; Huang, M.Y.; Deng, L.; Wu, Q.L.; Zeng, Y.X.; Shao, J.Y. MicroRNA miR-21 overexpression in human breast cancer is associated with advanced clinical stage, lymph node metastasis and patient poor prognosis. *RNA* **2008**, *14*, 2348–2360.
209. Li, X.; Liu, X.; Xu, W.; Zhou, P.; Gao, P.; Jiang, S.; Lobie, P.E.; Zhu, T. c-MYC-regulated miR-23a/24-2/27a cluster promotes mammary carcinoma cell invasion and hepatic metastasis by targeting Sprouty2. *J. Biol. Chem.* **2013**, *288*, 18121–18133.
210. Bernemann, C.; Hulsewig, C.; Ruckert, C.; Schafer, S.; Blumel, L.; Hempel, G.; Gotte, M.; Greve, B.; Barth, P.J.; Kiesel, L.; *et al*. Influence of secreted frizzled receptor protein 1 (SFRP1) on neoadjuvant chemotherapy in triple negative breast cancer does not rely on WNT signaling. *Mol. Cancer* **2014**, *13*, 174.
211. Du, W.W.; Fang, L.; Li, M.; Yang, X.; Liang, Y.; Peng, C.; Qian, W.; O'Malley, Y.Q.; Askeland, R.W.; Sugg, S.L.; *et al*. MicroRNA miR-24 enhances tumor invasion and metastasis by targeting PTPN9 and PTPRF to promote EGF signaling. *J. Cell Sci.* **2013**, *126 Pt 6*, 1440–1453.
212. Faget, J.; Sisirak, V.; Blay, J.Y.; Caux, C.; Bendriss-Vermare, N.; Menetrier-Caux, C. ICOS is associated with poor prognosis in breast cancer as it promotes the amplification of immunosuppressive CD4(+) T cells by plasmacytoid dendritic cells. *Oncoimmunology* **2013**, *2*, e23185.
213. Sreenath, A.S.; Kumar, K.R.; Reddy, G.V.; Sreedevi, B.; Praveen, D.; Monika, S.; Sudha, S.; Reddy, M.G.; Reddanna, P. Evidence for the association of synaptotagmin with glutathione S-transferases: Implications for a novel function in human breast cancer. *Clin. Biochem.* **2005**, *38*, 436–443.

214. Gu, Y.; Li, P.; Peng, F.; Zhang, M.; Zhang, Y.; Liang, H.; Zhao, W.; Qi, L.; Wang, H.; Wang, C.; *et al*. Autophagy-related prognostic signature for breast cancer. *Mol. Carcinog.* **2016**, *55*, 292–299.
215. Kovacevic, Z.; Fu, D.; Richardson, D.R. The iron-regulated metastasis suppressor, Ndrg-1: Identifi-cation of novel molecular targets. *Biochim. Biophys. Acta* **2008**, *1783*, 1981–1992.
216. Cormier, K.A.; Fournier, S.; O'Brien, J.; Tessier, P.; Robichaud, G.A. Role of s100a8 and s100a9 proteins on breast cancer aggressivity. *FASEB J.* **2009**, *23*, LB276.
217. Akhavantabasi, S.; Sapmaz, A.; Tuna, S.; Erson-Bensan, A.E. miR-125b targets ARID3B in breast cancer cells. *Cell Struct. Funct.* **2012**, *37*, 27–38.
218. Song, H.; Wu, C.; Wei, C.; Li, D.; Hua, K.; Song, J.; Xu, H.; Chen, L.; Fang, L. Silencing of DUSP6 gene by RNAi-mediation inhibits proliferation and growth in MDA-MB-231 breast cancer cells: An in vitro study. *Int. J. Clin. Exp. Med*. **2015**, *8*, 10481–10490.
219. Fang, W.B.; Jokar, I.; Zou, A.; Lambert, D.; Dendukuri, P.; Cheng, N. CCL2/CCR2 chemokine signaling coordinates survival and motility of breast cancer cells through Smad3 protein- and p42/44 mitogen-activated protein kinase (MAPK)-dependent mechanisms. *J. Biol. Chem.* **2012**, *287*, 36593–36608.
220. Sanchez-Barrena, M.J.; Vallis, Y.; Clatworthy, M.R.; Doherty, G.J.; Veprintsev, D.B.; Evans, P.R.; McMahon, H.T. Bin2 is a membrane sculpting N-BAR protein that influences leucocyte podosomes, motility and phagocytosis. *PLoS ONE* **2012**, *7*, e52401.
221. Campone, M.; Valo, I.; Jezequel, P.; Moreau, M.; Boissard, A.; Campion, L.; Loussouarn, D.; Verriele, V.; Coqueret, O.; Guette, C. Prediction of Recurrence and Survival for Triple-Negative Breast Cancer (TNBC) by a Protein Signature in Tissue Samples. *Mol. Cell. Proteom.* **2015**, *14*, 2936–2946.
222. Qie, S.; Chu, C.; Li, W.; Wang, C.; Sang, N. ErbB2 activation upregulates glutaminase 1 expression which promotes breast cancer cell proliferation. *J. Cell. Biochem.* **2014**, *115*, 498–509.
223. Yan, X.; Chen, X.; Liang, H.; Deng, T.; Chen, W.; Zhang, S.; Liu, M.; Gao, X.; Liu, Y.; Zhao, C.; *et al*. miR-143 and miR-145 synergistically regulate ERBB3 to suppress cell proliferation and invasion in breast cancer. *Mol. Cancer* **2014**, *13*, 220.
224. Feng, Q.; Zhang, Z.; Shea, M.J.; Creighton, C.J.; Coarfa, C.; Hilsenbeck, S.G.; Lanz, R.; He, B.; Wang, L.; Fu, X.; *et al*. An epigenomic approach to therapy for tamoxifen-resistant breast cancer. *Cell Res.* **2014**, *24*, 809–819.
225. Friedrichs, K.; Ruiz, P.; Franke, F.; Gille, I.; Terpe, H.J.; Imhof, B.A. High expression level of alpha 6 integrin in human breast carcinoma is correlated with reduced survival. *Cancer Res.* **1995**, *55*, 901–906.
226. Adem, C.; Soderberg, C.L.; Hafner, K.; Reynolds, C.; Slezak, J.M.; Sinclair, C.S.; Sellers, T.A.; Schaid, D.J.; Couch, F.; Hartmann, L.C.; *et al*. ERBB2, TBX2, RPS6KB1, and MYC alterations in breast tissues of BRCA1 and BRCA2 mutation carriers. *Genes Chromosomes Cancer* **2004**, *41*, 1–11.
227. Stephens, P.J.; McBride, D.J.; Lin, M.L.; Varela, I.; Pleasance, E.D.; Simpson, J.T.; Stebbings, L.A.; Edkins, S.; Mudie, L.J.; Greenman, C.D.; *et al*. Complex landscapes of somatic rearrangement in human breast cancer genomes. *Nature* **2009**, *462*, 1005–1010.
228. Pandey, A.K.; Zhang, Y.; Zhang, S.; Li, Y.; Tucker-Kellogg, G.; Yang, H.; Jha, S. TIP60-miR-22 axis as a prognostic marker of breast cancer progression. *Oncotarget* **2015**, *6*, 41290–41306.
229. Pennanen, P.T.; Sarvilinna, N.S.; Toimela, T.; Ylikomi, T.J. Inhibition of FOSL1 overexpression in antiestrogen-resistant MCF-7 cells decreases cell growth and increases vacuolization and cell death. *Steroids* **2011**, *76*, 1063–1068.
